# Global DNA Hypomethylation Prevents Consolidation of Differentiation Programs and Allows Reversion to the Embryonic Stem Cell State

**DOI:** 10.1371/journal.pone.0052629

**Published:** 2012-12-27

**Authors:** Christine S. Schmidt, Sebastian Bultmann, Daniela Meilinger, Benedikt Zacher, Achim Tresch, Kerstin C. Maier, Christian Peter, Dietmar E. Martin, Heinrich Leonhardt, Fabio Spada

**Affiliations:** 1 Department of Biology II, Ludwig Maximilians University Munich, Planegg-Martinsried, Germany; 2 Gene Center, Department of Biochemistry, Ludwig Maximilians University Munich, Munich, Germany; 3 Center for Integrated Protein Science Munich, Ludwig Maximilians University Munich, Munich, Germany; 4 Institute for Genetics, Botanical Institute, University of Cologne, Cologne, Germany; 5 Department for Computational Biology, Max Planck Institute for Plant Breeding Research, Cologne, Germany; 6 Sir William Dunn School of Pathology, University of Oxford, Oxford, United Kingdom; Michigan State University, United States of America

## Abstract

DNA methylation patterns change dynamically during mammalian development and lineage specification, yet scarce information is available about how DNA methylation affects gene expression profiles upon differentiation. Here we determine genome-wide transcription profiles during undirected differentiation of severely hypomethylated (*Dnmt1^−/−^*) embryonic stem cells (ESCs) as well as ESCs completely devoid of DNA methylation (*Dnmt1^−/−^*;*Dnmt3a^−/−^*;*Dnmt3b^−/−^* or TKO) and assay their potential to transit in and out of the ESC state. We find that the expression of only few genes mainly associated with germ line function and the X chromosome is affected in undifferentiated TKO ESCs. Upon initial differentiation as embryoid bodies (EBs) wild type, *Dnmt1^−/−^* and TKO cells downregulate pluripotency associated genes and upregulate lineage specific genes, but their transcription profiles progressively diverge upon prolonged EB culture. While Oct4 protein levels are completely and homogeneously suppressed, transcription of *Oct4* and *Nanog* is not completely silenced even at late stages in both *Dnmt1^−/−^* and TKO EBs. Despite late wild type and *Dnmt1^−/−^* EBs showing a much higher degree of concordant expression, after EB dissociation and replating under pluripotency promoting conditions both *Dnmt1^−/−^* and TKO cells, but not wild type cells rapidly revert to expression profiles typical of undifferentiated ESCs. Thus, while DNA methylation seems not to be critical for initial activation of differentiation programs, it is crucial for permanent restriction of developmental fate during differentiation.

## Introduction

Embryonic stem cells (ESCs) are pluripotent cells derived from the naïve epiblast of preimplantation blastocysts. Under appropriate conditions they can self renew indefinitely in the pluripotent state, as well as differentiate into any embryonic lineage, including germ cells, both *in vitro* and upon reintroduction in host embryos [1]. These properties make ESCs a powerful and popular model to investigate the molecular bases of pluripotency and lineage commitment. Indefinite self renewal of mouse ESCs is sustained by LIF/JAK/Stat3, PI3K/Akt and Wnt singalling as well as suppression of the FGF/Erk and GSK3 pathways [2]–[11]. These conditions support the expression of master transcriptional regulators of pluripotency, including Oct4, Nanog and Sox2 [12]–[15]. These transcription factors establish a core network that, in cooperation with epigenetic modifiers, non coding RNAs and the c-Myc transcriptional network, orchestrates the pluripotency expression program and suppresses differentiation programs [16]–[18] (reviewed in [19], [20]). Recent data suggest that the same core pluripotency factors also play crucial roles in initial cell fate choices. Differentiation signals directly modulate Oct4 and Sox2 protein levels, leading to changes in their genome wide binding profiles and thus initiating lineage selection without prior activation of lineage specification factors [21].

By indexing chromatin states through DNA and histone modification, epigenetic factors ensure stable propagation of transcription programs and thus contribute to cell identity. At the same time, epigenetic marks are typically reversible and the enzymatic systems that set and erase them respond directly or indirectly to environmental signals, providing the necessary plasticity for the dynamic changes of transcription programs required for progressive differentiation. Although many epigenetic factors and chromatin remodelers have a role in stabilizing the pluripotent ESC state (i.e., sustained self renewal and absence of spontaneous differentiation), most are actually not strictly required for its establishment and/or maintenance (as opposed to *bona fide* core pluripotency factors). This property and the demonstration that ESC self renewal is minimally dependent on extrinsic signaling have led to the concept that the pluripotent state of naïve epiblast and ESCs represents a ground proliferative state relatively independent from epigenetic regulation [22], [23]. In contrast, most epigenetic regulators are required for proper execution of transcriptional programs driving lineage commitment and progression of differentiation [20].

In mammals DNA methylation plays major roles in the control of gene expression during development and differentiation [24]–[26]. The importance of DNA methylation for proper development is underscored by the embryonic lethal phenotypes of mice lacking major DNA methyltransferases (Dnmts) [27]–[29]. These as well as many other studies have established that Dnmt3a and Dnmt3b, together with the catalytically inactive co-factor Dnmt3L, set DNA methylation patterns during embryogenesis and gametogenesis, while Dnmt1 is mainly responsible for maintaining these patterns through cell replication [30]–[34]. However, further studies have shown that Dnmt3 enzymes are also required for long term maintenance of DNA methylation patterns [35]–[38] and for their dynamic modulation in processes other than development [39], [40]. In embryos with homozygous inactivation of single *Dnmt* genes as well as in double Dnmt3a and 3b null (Dnmt3 DKO) embryos development arrests well after gastrulation, clearly showing that these enzymes are dispensable for the formation of naïve epiblast [28], [29]. In addition, corresponding Dnmt null ESCs can be readily derived from blastocysts or by direct gene targeting even in the case of triple knockout (TKO) of all catalytically active Dnmts (Dnmt1, 3a and 3b) [28], [29], [41]. Dnmt1 null ESCs have about 20% residual genomic methylation, mostly located in repetitive sequences [28], [36], [42]. TKO ESCs exhibit complete loss of genomic methylation, clearly showing that neither Dnmts nor DNA methylation are required for survival and self renewal of ESCs. Restoring Dnmt1 expression rescues the ability of Dnmt1 null ESCs to form teratomas, contribute to chimeras and complement tetraploid embryos [42], [43]. Analogously, the ability to form teratomas is restored in Dnmt3 DKO ESCs upon expression of either Dnmt3 protein [36]. Therefore, functional pluripotency of ESCs is not permanently compromised by the loss of either Dnmt1 or Dnmt3 proteins and consequent loss of DNA methylation.

Previous studies pointed to a very limited developmental potential of Dnmt1 null ESCs and to impaired survival or proliferation of their differentiated progeny [28], [44], [45]. Other studies showed that, in contrast to wt counterparts, Dnmt1 null ESCs differentiated as embryoid bodies (EBs) express high levels of trophoblast markers and, under appropriate conditions, can be differentiated into trophoblast derivatives [45], [46]. Similarly, it has been reported that TKO ESCs exhibit a growth defect and increased apoptosis upon EB differentiation and that cells from TKO nuclear transfer embryos aggregated with wt embryos mostly contribute to extraembryonic tissues, in addition to [41], [47]. However, in the latter study a few TKO cells were detected in the embryo proper till an early postgastrulation stage (E8.5), but their identity was not defined. Thus, it is not clear to what extent globally hypomethylated cells are able to commit to and progress along definitive embryonic lineages.

To investigate how DNA methylation contributes to differentiation programs, we subjected wt, *Dnmt1^−/−^* and TKO ESCs to undirected differentiation as EBs. Our results clearly show that hypomethylated cells initiate differentiation programs by downregulating pluripotency genes and upregulating markers of differentiated lineages. In particular, we show a previously unappreciated progression of differentiation programs in *Dnmt1^−/−^* EBs. However, by dissociating late EBs and replating their cells under culture conditions that promote pluripotency, we found that hypomethylated cells fully revert to the undifferentiated ESC state, indicating that DNA methylation is crucial for canalization of developmental fate during differentiation.

## Results

### Incomplete silencing of pluripotency gene transcription upon differentiation of globally hypomethylated ESCs

**Figure 1:**
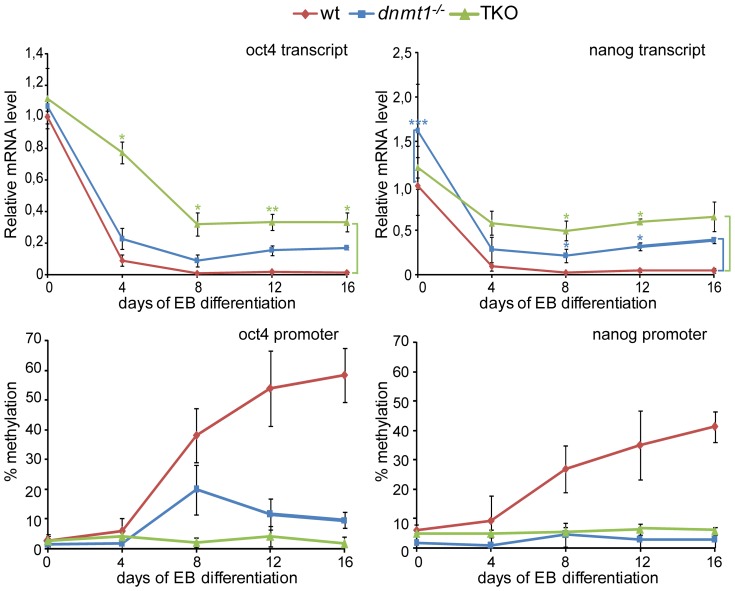
Partial silencing of *oct4* and *nanog* in *Dnmt1^−/−^* and TKO EBs. Oct4 and Nanog transcript levels (upper panels) and respective promoter methylation (lower panels) during differentiation of wt, *Dnmt1^−/−^* and TKO ESCs as EBs. Transcript levels were measured by RT-qPCR and are shown relative to wt ESCs (day 0). DNA methylation levels were determined by bisulfite conversion followed by PCR and pyrosequencing. Five and 4 CpG sites in the proximal promoter regions of *oct4* and *nanog*, respectively, were analyzed and respective values were averaged. Mean values and standard errors of 2 and 3 biological replicates are shown for DNA methylation and expression analyses, respectively. Asterisks indicate significance levels: * p<0.05; ** p<0.001; *** p<0.0001 (Student t-test).

To gain insights into the specific role of DNA methylation in silencing pluripotency associated genes during differentiation, we generated EBs from wt, *Dnmt1^−/−^* and TKO ESCs and analyzed transcript levels as well as promoter DNA methylation of the pluripotency genes *Oct4* (also known as *Pou5f1*) and *Nanog* (Fig. 1). Interestingly, wt and *Dnmt1^−/−^* EBs showed a drastic down regulation of both genes already by day 4, whereas DNA methylation of the respective promoters could be detected only by day 8. The observation that initial silencing of *Oct4* precedes methylation of its promoter is consistent with previous work showing recruitment of Dnmt3a by the GCNF-MBD3 complex to the *Oct4* promoter in advance of detectable DNA methylation [49]–[52]. To investigate the dependence of silencing and promoter methylation of *Oct4* and *Nanog* on the catalytic activity of Dnmt1 during differentiation we established clones of *Dnmt1^−/−^* ESCs stably expressing GFP fusions of wt Dnmt1 (GFP-Dnmt1^wt^) or a catalytically inactive single point mutant (GFP-Dnmt1^C1229W^) [48] and subjected them to the same differentiation procedure as above (Fig. S1). While silencing and promoter methylation of *Oct4* and *Nanog* in *Dnmt1^−/−^* EBs were largely restored to the levels present in wt ESCs by expression of GFP-Dnmt1^wt^, they were essentially unchanged in *Dnmt1^−/−^* EBs expressing GFP-Dnmt1^C1229W^. This shows that reduced promoter methylation and silencing of *Oct4* and *Nanog* in *Dnmt1^−/−^* EBs largely depend on the lack of Dnmt1 catalytic activity.

Consistent with an earlier report [47], TKO EBs also showed initial reduction of Nanog transcript levels, albeit more limited than in wt and *Dnmt1^−/−^* EBs (Fig. 1A). In contrast, *Oct4* silencing was remarkably delayed in TKO EBs. Importantly, in both *Dnmt1^−/−^* and TKO EBs reduction of Oct4 and Nanog transcript levels was partial as compared to wt EBs. Significant residual levels of both Oct4 and Nanog transcripts were detectable even in late mutant EBs and correlated with the extent of *Dnmt* gene deletions as well as with relative promoter methylation levels in the case of *Oct4* (Fig. 1), indicating that Dnmt3a and 3b contribute to the intermediate levels of *Oct4* and *Nanog* silencing in *Dnmt1^−/−^* EBs.

Taken together, these results reveal that promoter methylation is dispensable for initial silencing of *Oct4* and *Nanog*, but necessary for complete extinction of their transcriptional activity during differentiation.

### Complete and uniform downregulation of Oct4 protein levels in hypomethylated EBs

**Figure 2:**
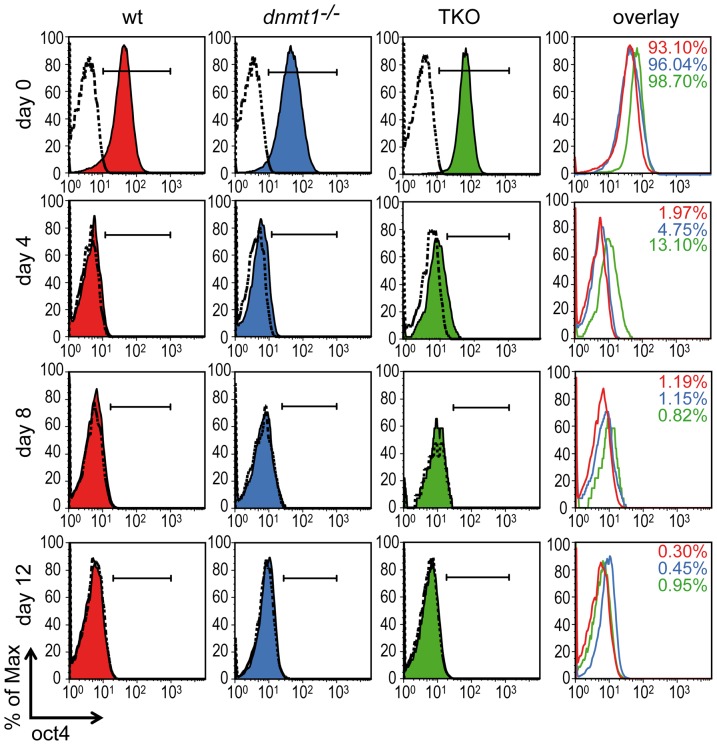
Uniform downregulation of Oct4 protein levels during EB differentiation. Undifferentiated ESCs colonies (day 0) and EBs at indicated stages of differentiation (day 4–12) were dissociated into single cells, stained with an Oct4 specific antibody and subjected to FACS analysis. Shaded curves depict Oct4 intracellular staining. Dashed lines indicate isotype controls and horizontal bars represent individual gates for positive Oct4 signals. The overlay shows exclusively Oct4 positive signals for each cell line. Numbers in red, blue and green indicate the percentages of Oct4 positive cells in wt, *Dnmt1^−/−^* and TKO ESCs/EBs, respectively, after subtraction of background signal from isotype controls.

We then asked whether the residual Oct4 transcript levels lead to residual Oct4 protein in *dnmt1^−/−^* and TKO EBs and whether downregulation of Oct4 protein occurs homogenously in EBs or subpopulations of cells retain high Oct4 protein levels. To address this question, we followed Oct4 protein levels during EB formation by immunofluorescent staining after cell dissociation and FACS analysis (Fig. 2). All ESC lines displayed similar levels of Oct4 protein in the pluripotent state. After 4 days of differentiation, only about 2% of cells from wt EBs were Oct4 positive, but higher numbers of positive cells were detected in mutant EBs. As observed at the transcript level (Fig. 1), a substantial delay in downregulation was also found for Oct4 protein levels in TKO EBs as about 13% of cells were still Oct4 positive at day 4. Again *dnmt1^−/−^* EBs showed a less dramatic phenotype at day 4 as only 4.75% of their cells still contained detectable Oct4 protein. Surprisingly, regardless of genotype Oct4 positive cells were about 1% at day 8 and below 1% by day 12. At the latter time point Oct4 staining profiles showed no distinct cell subpopulation expressing high levels Oct4 protein and were indistinguishable among genotypes and from the background profiles of the antibody isotype control. We conclude that, despite incomplete extinction of *Oct4* transcription, the downregulation of Oct4 protein during differentiation is complete and uniform also in hypomethylated cells.

### DNA methylation is dispensable for activation of differentiation programs

**Figure 3:**
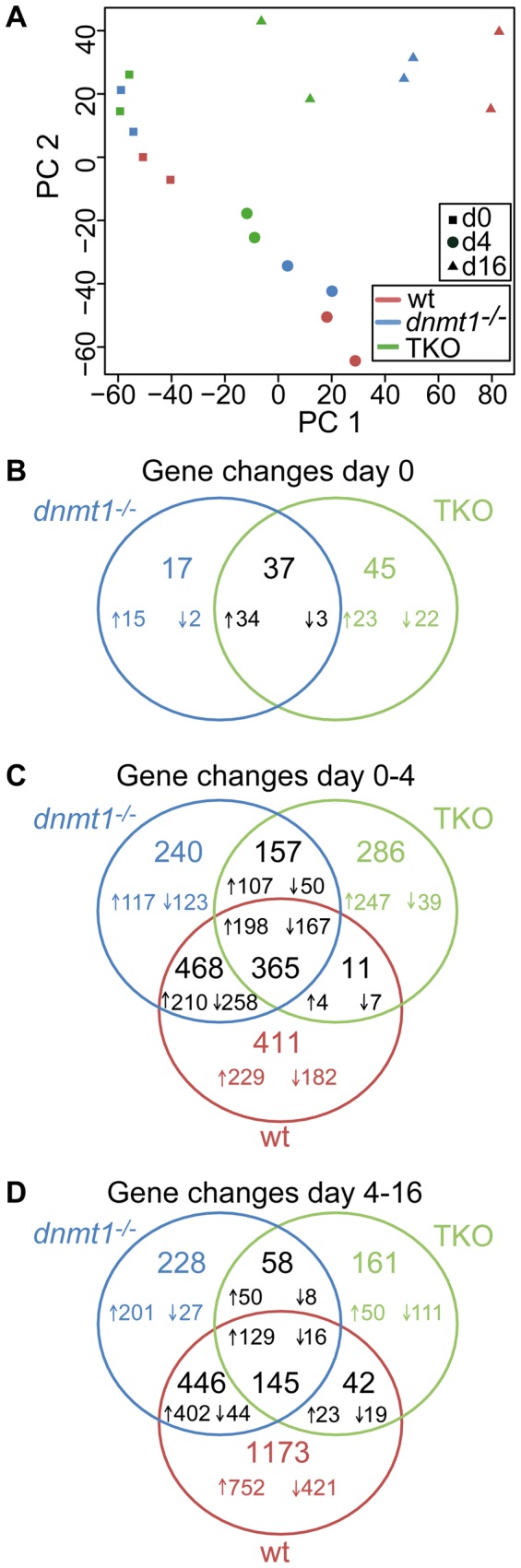
Genome-wide expression analysis of wt, *Dnmt1^−/−^* and TKO ESCs during differentiation as EBs. RNA samples from undifferentiated ESCs and respective EBs at day 4 and 16 of differentiation were subjected to microarray expression analysis. A) Two dimensional principal component analysis of independent biological duplicates per cell line per time point. B) Venn diagram of differentially expressed genes in *Dnmt1^−/−^* (blue circles) and TKO (green circles) ESCs with respect to wt ESCs. (C, D) Venn diagram of gene changes occurring between day 0–4 (C) and day 4–16 (D) in knockout and wt (red circles) EBs. Gene changes occurring during the two differentiation intervals in wt and mutant EBs were determined and compared. Larger numbers indicate total numbers of gene changes, whereas smaller numbers refer to up- (↑) and down- (↓) regulated genes in the respective sectors. Data were averaged from independent biological duplicates.

As we found that hypomethylated cells are capable of partially silencing the master pluripotency genes *Oct4* and *Nanog* as well as completely suppressing Oct4 protein levels, we next asked whether these cells are able to activate differentiation programs. To address this question, we performed genome-wide microarray expression analysis of undifferentiated wt, *Dnmt1^−/−^* and TKO ESCs and corresponding EBs at day 4 and 16 of differentiation in two independent experiments. Principal component analysis (PCA) showed that data points segregate according to day of differentiation and genotype (Fig. 3A). The three undifferentiated ESC lines were relatively closely clustered, suggesting that their expression profiles are very similar. However, principal component 1 provided a clear differentiation signature, as it increased continuously with EB culture time. This increase was less pronounced for the knockout genotypes, reflecting a crucial contribution of DNA methylation to the control of gene expression during differentiation. At both differentiation time points *Dnmt1^−/−^* EBs were at an intermediate distance along the median line between TKO and wt EBs, again emphasizing the less severe phenotype of *Dnmt1^−/−^* EBs compared to TKO EBs.

Using a two-fold cutoff and false discovery rate below 5%, we determined gene expression changes in undifferentiated *Dnmt1^−/−^* and TKO ESCs relative to wt ESC lines (Fig. 3B). In addition, we measured gene expression changes for each genotype between the undifferentiated ESC state and day 4 EBs (Fig. 3C), as well as between day 4 and day 16 EBs (Fig. 3D). Differentially expressed genes and their expression fold change are listed in Table S5 (d0), S6 (d0–4) and S7 (d4–16). As suggested by PCA, very few genes showed altered expression in undifferentiated hypomethylated ESCs (*Dnmt1^−/−^* 54, TKO 82). As DNA methylation is mostly considered to be involved in gene silencing [53], we reasoned that decreased expression in hypomethylated ESCs/EBs is most likely due to indirect effects. Therefore, we focused most of our analyses on upregulated genes in mutant ESCs/EBs. Gene ontology (GO) enrichment analysis of the 57 genes upregulated in TKO ESCs revealed categories involved in reproductive processes like oogenesis and spermatogenesis (Fig. S2A). In contrast, no enriched category could be identified for the 49 genes upregulated in *Dnmt1^−/−^* ESCs. However, in both mutant ESC lines, disregulated genes were frequently located on the X chromosome (*Dnmt1^−/−^* 13.1%; TKO 40.4%) and in *Dnmt1^−/−^* ESCs also on the Y chromosome (13.3%). Furthermore, in both mutant ESC lines about 60% of the upregulated transcripts are known to exhibit testis specific expression. These data are consistent with previous results from Fouse *et al.* [54], where a Dnmt3 DKO ESC line with constitutive knockdown of Dnmt1 was analyzed. Surprisingly though, only 60 out of 274 genes that we identified as differentially expressed in TKO ESCs were found disregulated also in the previous study. This may at least in part be due to the less stringent cutoff criteria used in Fouse *et al.* as when we compared the GO terms associated with disregulated genes, we found a more substantial overlap between the two studies (Fig. S2B).

In the first differentiation period (d0–4) changes in transcript levels were observed for approximately the same number of genes in wt (1255) and *Dnmt1^−/−^* EBs (1230), two thirds of which (833) changed expression concordantly in the two EB populations. In comparison, total gene expression changes in TKO EBs were roughly two thirds (819) of those in wt EBs, but the concerted changes (376) were only one third of them (Fig. 3C and D). Importantly, in the second differentiation period (d4–16) the expression levels of 1808 genes changed in wt EBs, while roughly half (879) and one fifth (406) of expression changes were detected in *Dnmt1^−/−^* and TKO EBs, respectively. With respect to the expression changes in wt EBs one third (593) and only one tenth (187) were actually concordant in *Dnmt1^−/−^* and TKO EBs, respectively. The initial high proportions of concomitant gene expression changes in mutant and wt EBs suggest that hypomethylated cells can activate differentiation programs to substantial extents. However, the further decrease of concordant gene expression changes seen between day 4 and 16 suggests that progression of differentiation programs in mutant EBs was impaired. This was particularly pronounced in TKO EBs, indicating that the presence of Dnmt3 proteins allows hypomethylated *Dnmt1^−/−^* EBs to execute differentiation programs to an extent that is more similar to that of wt EBs.

After the first four days of differentiation 365 genes showed similar expression changes in wt and mutant EBs (Fig. 3C). GO analysis of the 198 upregulated genes in this group revealed significant enrichment in categories related to developmental processes like anatomical structure development, system and organ development and cell differentiation (Fig. S3A). By contrast, the 167 genes that were downregulated in EBs of all three genotypes were significantly enriched in genes involved in stem cell maintenance and development as well as genes expressed in ESCs and embryonic germ cells (EGCs) (Fig. S3B and S3C). These data clearly show that all three ESC lines initiated differentiation programs by downregulating genes associated with pluripotent stem cell states and upregulating genes required for developmental processes.

### Differentiation programs progress further in *Dnmt1^−/−^* than in TKO EBs

Examination of gene changes in the second period of EB differentiation (day 4–16) revealed that only 145 genes were concordantly expressed in wt, *Dnmt1^−/−^* and TKO EBs. Enriched GO categories for the 129 concerted up regulated genes in all three cell lines were involved in lipid metabolic processes and were mainly expressed in extraembryonic tissues (Fig. S4). Importantly, substantially more genes were concomitantly regulated in wt and *Dnmt1^−/−^* EBs after both differentiation periods. In the first four days, in addition to the 365 genes with concordant expression changes in all three genotypes, 468 more genes were commonly regulated in wt and *Dnmt1^−/−^* EBs (Fig. 3C). From the 210 upregulated genes of this group the most highly enriched GO terms were associated with developmental processes, including cell differentiation and proliferation, tissue and organ development (Fig. S5), and Kegg pathway analysis indicated enrichment for genes involved in signaling pathways, including the Wnt and TGF-β pathways (not shown). Furthermore, the 258 genes that were commonly downregulated in day 4 wt and *Dnmt1^−/−^* EBs yielded 35 enriched GO categories including regulation of expression, metabolism and developmental processes (not shown). During the second differentiation period (day 4–16) 446 genes were also concordantly regulated in wt and *Dnmt1^−/−^* EBs, with 90% (402) upregulated genes (Fig. 3D). The latter genes were enriched in GO terms mainly associated with cell adhesion, hemostasis, organ development and metabolism (Fig. S6A) and are known to be expressed in various tissues like liver, plasma and kidney (Fig. S6C), while the few genes that were commonly downregulated (44) were related to GO categories of pattern specification and embryonic development (Fig. S6B). These observations reveal a previously unappreciated progression of differentiation programs in *Dnmt1^−/−^* EBs and show that *Dnmt1^−/−^* cells respond more to EB differentiation conditions than TKO cells.

**Figure 4:**
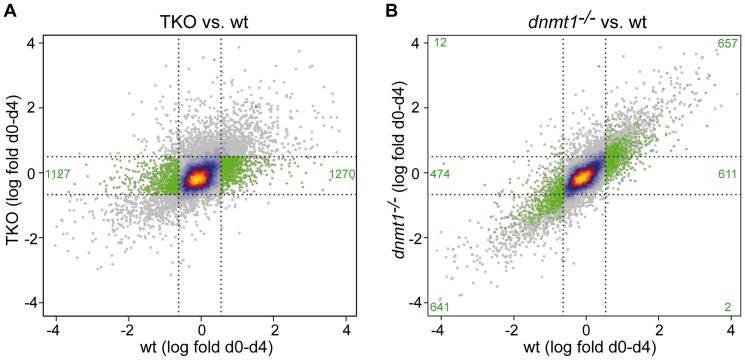
Genome-wide expression changes in early wt, *Dnmt1^−/−^* and TKO EBs. Scatter plots of genome-wide expression changes between day 0 and 4 in TKO (A) and *Dnmt1^−/−^* EBs (B) relative to wt EBs. Data points are heat-colored according to their local density. About 17% of 25528 analyzed genes changed expression level in wt EBs according to the thresholds indicated by the vertical dotted lines. Genes whose expression levels increased and decreased in wt but not in TKO EBs (“TKO non-responders”, parts of the corridor between the horizontal dotted lines to the left and right of the vertical dotted lines in A) are marked in green in both A and B and their numbers are reported in green in the respective sectors.

To gain a measure of the developmental potential of *Dnmt1^−/−^* and TKO EBs we compared their expression changes relative to wt EBs in greater detail and focused on changes occurring during the first four days of differentiation as TKO EBs seemed unable to progress substantially in the following 12 days (day 4–16). We plotted the expression changes in either TKO or *Dnmt1^−/−^* EBs relative to wt EBs for all genes (25528) (Fig. 4A and B). In this analysis we used a two-fold cutoff as well, but did not filter for false discovery rate, resulting in higher numbers of gene expression changes than in the case of Fig. 3C. Data points clustered much closer to the diagonal line in the plot showing expression changes in *Dnmt1^−/−^* relative to wt EBs (Fig. 4B), confirming the higher concordance of gene changes between these EB populations. We identified 2397 genes that do not respond to the differentiation conditions in TKO EBs (“non-responders”; green dots in Fig. 4A). By tracking these genes in the expression change plot for *Dnmt1^−/−^* versus wt EBs it becomes evident that more than half of these genes (1298 out of 2397) did respond similarly in *Dnmt1^−/−^* and wt EBs. This analysis also confirmed that in total 70% of gene expression changes were concordant between wt and *Dnmt1^−/−^* EBs.

The further progress of differentiation programs in *Dnmt1^−/−^* relative to TKO EBs suggests that expression of Dnmt3a and 3b proteins endows *Dnmt1^−/−^* cells with greater differentiation potential. To support this concept we analyzed Dnmt3a and 3b transcript levels in wt and *Dnmt1^−/−^* EBs after 4 and 16 days of culture. Consistent with previous immunofluorescence data on embryos [55] Dnmt3b transcripts progressively declined in wt EBs, while Dnmt3a transcripts first decreased in early EBs and then recovered at the later stage. As compared to wt EBs higher levels of Dnmt3b transcripts were detected in both early and late *Dnmt1^−/−^* EBs, while Dnmt3a transcript levels were the same and lower at day 4 and 16, respectively, (Fig. S7). Interestingly, *Dnmt3b* promoter activity was shown to be controlled by DNA methylation [56], which could explain the higher levels of Dnmt3b transcripts in hypomethylated *Dnmt1^−/−^* EBs. Thus, high Dnmt3b levels may contribute to the further progression of differentiation programs in *Dnmt1^−/−^* versus TKO EBs.

### Silencing of most bivalent genes during early EB differentiation does not require *de novo* methylation or Dnmt proteins

In mouse ESCs, approximately three thousand genes feature chromatin domains with frequent trimethylation of both lysines 4 and 27 in histone H3 (H3K4me3 andH3K27me3), marks usually found separately in chromatin with opposite transcriptional potential. These bivalent genes encode lineage specific factors and were shown to be transcriptionally poised for rapid activation upon differentiation [57], [58]. Interestingly, recent work showed that 93% of all bivalent promoters contain CpG islands and suggested a transition from the bivalent state to DNA methylation upon differentiation of ESCs to neural progenitors [59]. To test whether a similar switch occurs more generally in undirected EB differentiation conditions we applied the gene expression analysis shown above to bivalent and non-bivalent gene sets separately (Fig. S8). Despite the different numbers of genes, this revealed similar distributions for the separate gene sets as for all genes together, indicating that there is no general trend for genes which are bivalent in the ESC state to be silenced by DNA methylation or Dnmt protein-dependent mechanisms during early differentiation. Instead, a tendency could be observed for bivalent genes not to change their expression levels under EB differentiation conditions (i.e., to cluster more at the center of the plots in Fig. S8). This is expected due to the heterogeneous cell type composition in EBs, as bivalent genes are activated only in specific cell types and kept silent in all other lineages.

### Dnmts are required for silencing selected bivalent genes during differentiation

**Figure 5:**
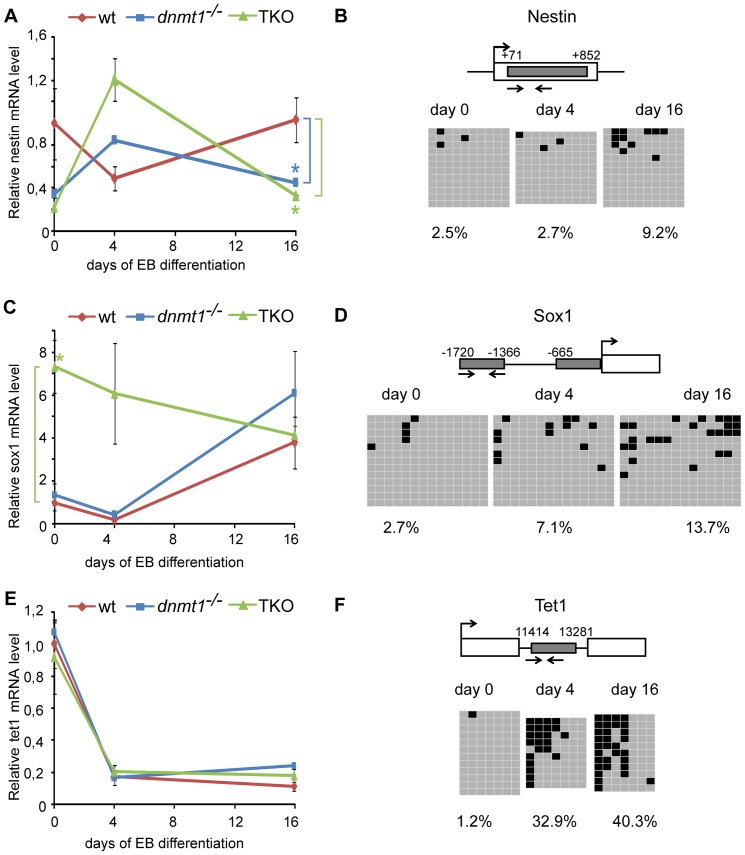
CpG island methylation and disregulated expression of selected bivalent genes in wt and mutant EBs. Transcript levels and CpG island methylation of bivalent genes *Nestin* (A,B) and *Sox1* (C,D) as well as the non-bivalent gene *Tet1* (E,F) in undifferentiated ESCs (day 0) and at day 4 and 16 of EB culture. (A,C,E) Transcript levels were measured by RT-qPCR in wt, *Dnmt1^−/−^* and TKO ESCs/EBs as indicated. Mean values and standard errors from three independent biological replicates are shown. All values are normalized to undifferentiated wt ESCs. Asterisks indicates the significance level p<0.05 (Student t-test). (B,D,F) DNA methylation analysis by bisulfite sequencing in wt ESCs/EBs. In the gene cartoons large arrows indicate transcriptional start sites (TSS), open rectangles represent exons, grey shaded rectangles represent CpG islands and numbers indicate the positions of their borders with respect to the TSS. Small arrows indicate the analyzed regions. In the panels grey and black squares indicate unmethylated and methylated CpG sites, respectively. Percentages of total CpG methylation within the analyzed regions/clones are reported at the bottom of each panel.

We also analyzed genes that were upregulated exclusively in *Dnmt1^−/−^* and/or TKO EBs as DNA methylation and/or Dnmt3 proteins may be critical for their repression. The 107 genes upregulated in both *Dnmt1^−/−^* and TKO EBs during the first differentiation period (Fig. 3C) were enriched in GO terms for developmental processes like system and organ development as well as anatomical structure development (Fig. S9A). Intriguingly, GO enrichment analysis revealed that several genes exclusively upregulated in either *Dnmt1^−/−^* or TKO EBs play a role in nervous system development (Fig. S9B and D) and, in the case of TKO EBs, many are known to be expressed in the brain (Fig. S9C). Upregulated genes in TKO EBs involved in neural differentiation included *Nestin*, *Hes6*, *Fapb7/Blbp*, *Otx2* and *Fzd3*, which have roles in neural tube patterning and neural progenitors [60]. Interestingly, *Nestin* and *Otx2* are bivalent in ESCs and may have been missed in our global analysis of bivalent gene expression in hypomethylated EBs (Fig. S8B). To investigate whether selected bivalent genes, including genes expressed in neural progenitors, are controlled by DNA methylation or Dnmt proteins we analyzed CpG island methylation of *Nestin* (all ectodermal progenitors) and *Sox1* (early neuroepithelial progenitors) during EB differentiation of wt cells and verified their expression in wt, *Dnmt1^−/−^* and TKO ESCs and EBs (Fig. 5 and Fig. S10). The same analyses were extended to *Fgf5* and *Brachyury* as representatives of non-neural bivalent genes (primitive ectoderm and early mesoderm, respectively) and *Tet1*, a non-bivalent gene that is rapidly silenced upon EB differentiation [61]. The expression profiles of *Nestin*, *Sox1, Fgf5* and *Brachury* were altered in mutant ESCs/EBs, although to variable extents and at different time points. Interestingly, among these bivalent genes an increase in CpG island methylation during differentiation was seen only for *Nestin* and *Sox1* (Fig. 5B and D). The distribution of methylated sites in the different clones obtained by bisulfite sequencing is consistent with increased methylation of these genes in selected lineages. In contrast, all clones in the *Tet1* bisulfite analysis showed increased methylation, although Tet1 transcript levels were not affected in mutant EBs (Fig. 5C). These data are consistent with a requirement of Dnmts for silencing of selected bivalent genes.

### Hypomethylated cells isolated from late EBs revert to the undifferentiated state

**Figure 6:**
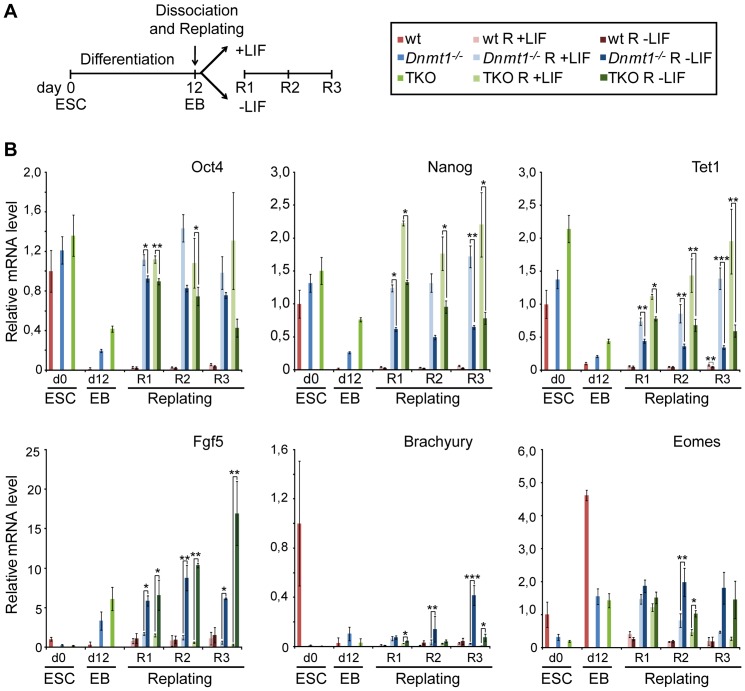
LIF signaling induces reversion of cells from *Dnmt1^−/−^* and TKO EBs to the ESC state. (A) Overview of experimental set up. Day 12 EBs were dissociated and their cells were plated and further cultured for three days (R1–3) in the presence or absence of LIF. (B) Transcript levels of pluripotency associated genes *Oct4*, *Nanog* and *Tet1*, as well as differentiation marker genes *Fgf5*, *Brachyury* and *Eomes* were determined by RT-qPCR in undifferentiated ESCs (day 0), day 12 EBs and 1, 2 and 3 days after replating (R1–3). Wild type, *Dnmt1^−/−^* and TKO samples are represented in shades of red, blue and green, respectively, as indicated in the box at the upper right corner. Mean values relative to wt ESCs (day 0) and standard errors are from three independent biological replicates. Asterisks indicate significance levels: * p<0.05; ** p<0.001; *** p<0.0001 (Student t-test).

We showed that Oct4 protein levels were completely downregulated in hypomethylated EBs, although silencing of *Oct4* and *Nanog* transcription was partial relative to wt EBs. At the same time hypomethylated EBs were able to activate differentiation programs, but their progression was altered to an extent that correlated with the presence of Dnmt3 proteins. Thus, we wondered whether cells from *Dnmt1^−/−^* and TKO EBs could revert to the undifferentiated state in conditions that promote pluripotency. To test this we dissociated 12 days old wt, *Dnmt1^−/−^* and TKO EBs into single cells, plated equal cell numbers in the presence or absence of LIF and analyzed the expression of pluripotency associated genes as well as markers of differentiated lineages after one (R1), two (R2) or three days (R3; Fig. 6). We chose 12 days old EBs as our results clearly demonstrated that at this stage Oct4 protein levels were homogenously down regulated in all living cells regardless of genotype (Fig. 2). As expected, upon replating wt cells maintained a heterogeneous, differentiated morphology (not shown) and their transcript levels of pluripotency associated factors as well as Fgf5 and Brachury showed essentially no response regardless of the presence or absence of LIF. Indeed, *Fgf5* and *Brachury* are only transiently expressed in the embryonic primitive ectoderm and early mesoderm and in EBs they peaked at time points before day 12 (not shown). Instead, *Eomes* is expressed in trophoectodermal derivatives and showed drastic downregulation upon EB dissociation, pointing to a role of cell-to cell contacts in promoting its expression. In contrast to cells from wt EBs, cells dissociated from *Dnmt1^−/−^* and TKO EBs rapidly formed colonies with the characteristic morphology of undifferentiated ESCs (not shown) and upregulated transcript levels of pluripotency associated factors within 24 h. Strikingly, in dissociated Dnmt null cells upregulation of *Oct4*, *Nanog* and *Tet1* was observed also upon culture without LIF, pointing to a role of cell-to-cell contact in restraining the expression of these genes. However, Oct4, Nanog and Tet1 transcript levels were significantly lower in the absence than in the presence of LIF and in TKO cells they decreased to similar levels as in corresponding day 12 EBs. In dissociated *Dnmt* mutant cells cultured with LIF not only were Oct4, Nanog and Tet1 transcripts upregulated to higher levels already 24 h after replating, but their levels were maintained or even increased in the following days so that at least by the third day they were similar to the levels originally found in the respective undifferentiated ESC cultures. In dissociated *Dnmt* mutant cells the expression of differentiated lineage markers decreased or increased depending on the presence or absence of LIF, respectively. Again, expression levels similar to those originally present in the undifferentiated ESC cultures were reached in the presence of LIF, while in the absence of LIF they were the same as in corresponding day 12 EBs (Eomes) or higher (Fgf5 and Brachury). To validate these data we subjected our *Dnmt1^−/−^* ESC lines stably complemented with GFP-Dnmt1^wt^ and GFP-Dnmt1^C1229W^ to the same procedure (Fig. S11). Complementation with the catalytically inactive GFP-Dnmt1^C1229W^ resulted in transcript levels of *Oct4*, *Nanog* and *Tet1* very similar to those in parental *Dnmt1^−/−^* cells/EBs at every stage. In contrast and as expected, stable complementation of *Dnmt1^−/−^* cells/EBs with GFP-Dnmt1^wt^ led to drastic silencing of *Oct4*, *Nanog* and *Tet1* in EBs, that, as in wt cells/EBs, was maintained after EB dissociation and replating, regardless of the presence or absence of LIF. Transcript levels of *Fgf5*, *Brachury* and *Eomes* in *Dnmt1^−/−^* cells/EBs stably complemented with GFP-Dnmt1^wt^ and GFP-Dnmt1^C1229W^ also showed similar dynamics to *Dnmt1^−/−^* and wt cells/EBs, respectively, with some deviation likely due to constitutive (unregulated) expression of GFP-Dnmt1 constructs.

These results clearly show that cells isolated from 12 days old *Dnmt1^−/−^* and TKO EBs respond to the combined action of dissociation and LIF stimulation by reverting to the undifferentiated ESC state. This result is particularly striking in the case of *Dnmt1^−/−^* EBs where differentiation programs are relatively advanced as shown by high concordance of expression profiles with those from wt EBs. In addition, the DNA methyltransferase activity, rather than a non-catalytic function of Dnmt1, is required to suppress the ability of cells from *Dnmt1^−/−^* EBs to revert to the ESCs state upon dissociation and exposure to LIF. Together these data underscore a fundamental role of DNA methylation in restricting developmental plasticity by locking transcriptional states during differentiation.

## Discussion

The role of DNA methylation in controlling transcription programs during mammalian development and lineage specification has been mainly inferred from genomic methylcytosine profiles in a limited selection of cell types and developmental stages, while little information is available about how DNA methylation and Dnmts actually affect transcription during differentiation. In particular, expression data from differentiated progeny of globally hypomethylated ESCs lacking specific Dnmts are very scarce. This is likely in relation to reports of limited survival or proliferation of Dnmt1 null and TKO cells upon differentiation [28], [44], [45], [47]. Consistent with this previous work, we found that the average size of Dnmt1 null and especially TKO EBs is reduced as compared to that of wt EBs. However, we could maintain cultures of these mutant EBs for at least 24 days. EB formation is an undirected differentiation model supporting the specification of a broad range of cell fates and thus commonly used to asses developmental potential. Using this system we found that Dnmt1 null and TKO EBs exhibit residual transcription of pluripotency master regulators *Oct4* and *Nanog* even after 16 days of culture. However, FACS analysis showed that by day 8 Oct4 protein is uniformly downregulated in all the cells of these mutant EBs to the same basal levels as in wt EBs, suggesting homogeneous exit from the ESC state. This is supported by the relatively high concordance of genome-wide transcription changes in mutant relative to wt EBs after 4 days of differentiation, including downregulation of additional genes associated with pluripotent stem cell states and upregulation of factors related to differentiated lineages. However, by day 16 gene expression profiles in TKO EBs reveal a high degree of divergence from those in wt EBs, while expression changes in Dnmt1 null EBs are still concordant with one third of all changes in wt EBs. On the one hand this represents a previously unappreciated progression of transcription programs in differentiated Dnmt1 null cells, on the other hand it reflects substantially impaired developmental potential in TKO as well as Dnmt1 null cells. In this regard it is worth considering that while the genome of TKO cells is virtually devoid of methylation, the 20% residual genomic methylcytosine in *Dnmt1^−/−^* ESCs is mainly restricted to repetitive sequences [28], [36], [42]. Yet our analysis reveals a remarkable difference between gene expression profiles of *Dnmt1^−/−^* and TKO cells in response to differentiation conditions. These differences must ultimately depend on the action of Dnmt3a and 3b, either through sustained *de novo* methylation of repetitive sequences, indirect effects or mechanisms independent from their catalytic activity. Interestingly, ESCs lacking both Ring1b and Eed, and thus functional Polycomb repressive complexes (PRC) 1 and 2, have also been recently shown to retain the ability to self renew, yet they fail to properly execute differentiation programs and silence repetitive sequences, including endogenous retroviral elements (ERVs) [23]. In particular, ESCs lacking both PRC1 and 2 show twice the number of derepressed genes as ESCs lacking either complex, where little derepression of ERVs is observed, leading to the proposal that repetitive sequences serve as a platform for gene silencing by PRC complexes. This is reminiscent of the higher number of disregulated genes in TKO versus Dnmt1 null EBs (roughly two fold at day 4) and of substantial residual methylation of repetitive sequences in Dnmt1 null cells. It is therefore tempting to speculate that methylation of repetitive sequences may also serve as a platform for gene silencing upon differentiation. Thus, there seem to be striking parallels between the roles and possible modes of action of the DNA methylation and Polycomb systems in contributing to cell identity and developmental potential.

Upon initial lineage commitment some bivalent domains may remain such to perpetuate the poised state for later differentiation events, while most are thought to resolve by loss of H3K27me3 for transcriptional activation or loss of H3K4me3 or both H3 marks accompanied by gain of DNA methylation for definitive repression as it has been proposed in the case of neural commitment [59], [62]. If DNA methylation represented a general mechanism for permanent silencing of bivalent genes in non-expressing lineages most of these genes would be expected to be disregulated in Dnmt1 null or at least TKO EBs, which we find not to be the case. However, we show that the bivalent genes *Nestin* and *Sox1*, which are both expressed in early neuroepithelial progenitors, do gain methylation at their CpG islands in a subset of wt differentiated cells and are disregulated in the absence of Dnmts. This suggests that CpG island methylation contributes to silencing of *Nestin* and *Sox1* in specific lineages. By contrast, we did not detect CpG island methylation for the bivalent genes *Fgf5* and *Brachyury* (expressed in primitive ectoderm and early mesoderm, respectively), which were also disregulated in mutant EBs. These data are consistent with an involvement of Dnmts in mediating stable silencing of selected bivalent genes during differentiation through either direct or indirect mechanisms. Further work is required to establish whether CpG island methylation is the preferred silencing mechanism for genes with function in the neuroectodermal lineage.

Regardless of bivalent nature, we also note that genes with roles in neural fate specification are frequently upregulated in TKO EBs. This is consistent with a recent report showing involvement of DNMT3B in controlling neural selector genes upon neural differentiation of human ESCs [63]. In this case knockdown of DNMT3B determined loss of H3K27me3 and EZH2 occupancy (the H3K27 methyltransferase component of PRC2) at promoters of affected genes, but not DNA methylation, suggesting once more a role of a Dnmt in recruiting other silencing factors (in this case DNMT3B recruiting PRC2).

We show that Dnmt1 null and TKO cells isolated from 12 days old EBs can fully and rapidly revert to the undifferentiated ESC state when plated in pluripotency promoting conditions. It is highly unlikely that this is due to a subpopulation of undifferentiated cells within day 12 Dnmt1 null and TKO EBs as our FACS analysis of Oct4 protein profiles shows no distinct subpopulation of living cells dissociated from these mutant EBs that express levels above background (as defined by antibody isotype control; Fig. 2). Importantly, the same background profiles are found in cells from wt EBs, which do not respond to replating in the presence of LIF. In addition, our genome wide expression analysis shows that Dnmt deficient EBs, especially *Dnmt1^−/−^* EBs, upregulate differentiation programmes to a substantial extent. However, in day 12 *Dnmt* mutant EBs transcription of *Oct4* and *Nanog* is not completely silenced and upon dissociation and exposure to LIF both genes are rapidly and fully reactivated. Interestingly, increased transcriptional activity of *Oct4*, *Nanog* and *Tet1* is also seen upon replating of cells from *Dnmt* mutant EBs in the absence of LIF. Surprisingly, during the following two days the levels of Oct4, Nanog and Tet1 transcripts in replated TKO cells gradually return to those present in corresponding day 12 EBs, while they are maintained in replated *Dnmt1^−/−^* cells. The reason for this difference between Dnmt1 null and TKO cells is unclear. In this regard we note that genes concordantly upregulated in wt and *Dnmt1^−/−^* EBs between 4 and 16 days of differentiation show highest enrichment for GO terms associated with cell adhesion (p<1e^−6^; Fig. S6A). Together these results suggest that cell-to-cell contact within EBs contributes to silencing of *Oct4*, *Nanog* and *Tet1* and that this repressive effect is released upon dissociation of hypomethylated EBs. However, the transcriptional response of *Oct4*, *Nanog* and *Tet1* to the release of cell-to-cell contact is subordinate to silencing by promoter methylation, as cells from wt EBs do not reactivate these genes upon replating, regardless of LIF stimulation. Thus, although additional epigenetic pathways are known to corepress *Oct4* and *Nanog* and may respond to cell adhesion conditions, our data show that DNA methylation is crucial for complete and permanent extinction of *Oct4* and *Nanog* transcription and thus enforces canalization of developmental fate upon differentiation.

Global inhibition of Dnmt activity was shown to facilitate reprogramming of differentiated somatic cells to pluripotency [64], [65]. However, this approach may have undesired effects, especially in the case of mechanism based inhibitors that lead to the formation of covalent and potentially mutagenic Dnmt-DNA adducts. Our observation that differentiated cells lacking only Dnmt1 efficiently revert to the ESC state suggests that transient and specific inhibition of Dnmt1 activity may be sufficient to promote conversion of differentiated cell types to the pluripotent state. However, this could also be counteracted by death of not yet dedifferentiated cells as Dnmt1 ablation in differentiated cells was shown to trigger apotosis at least in part mediated by p53 [66]. At the same time functional p53 inactivation has been shown to increase the efficiency of iPSC derivation by overcoming proliferative senescence of differentiated cells [67]–[71]. Thus, combining transient functional inactivation of Dnmt1 and p53 may have a synergistic effect on the reprogramming efficiency. Indeed, p53 inactivation may favor rapid passive demethylation by increasing proliferation rates and at the same time it may prevent death of not yet dedifferentiated cells.

In conclusion, our results underscore a critical role of DNA methylation and Dnmts in restricting developmental potential by permanently sealing transcriptionally silent states, as in the case of the pluripotency genes *Oct4* and *Nanog* and genes involved in the neuroectodermal lineage. In addition, our results lend support to transient Dnmt1 inhibition as an approach for improved reprogramming of differentiated cells to the pluripotent state, which in turn suggests functional p53 inactivation as a potentially synergistic strategy.

## Materials and Methods

### Cell and EB culture

The mouse wt, *dnmt1^−/−^* and TKO J1 ESC lines and respective EBs were cultured as described previously [61]. For stable complementation, *dnmt1^−/−^* ESCs were transfected with pCAG-IRES-blast vectors with inserts encoding GFP-Dnmt1^wt^ or GFP-Dnmt1^C1229W^ fusions [72]. Stable clones were selected either by culture in the presence of blasticidin or by repeated FACS sorting. Clones used in the present work were further selected as to express steady state levels of GFP-Dnmt1 fusions similar to those of native Dnmt1 in wt ESCs (Fig. S1A). For replating of cells from EBs, EBs were washed three times with PBS and dissociated to single cells by repeated cycles of Accutase treatment (PAA Laboratories GmbH) at 37°C. Dissociated cells were diluted in EB medium (ES cell medium without LIF), centrifuged and resuspended in fresh EB medium. Equal numbers of cells were then seeded in duplicate on gelatin-coated plates and LIF (ESGRO, Millipore) was added to one of the duplicate plates to a final concentration of 1000 U/ml.

### DNA and RNA isolation

Genomic DNA (gDNA) and total RNA from ESCs and EBs were extracted using either the NucleoSpin Triprep Kit (Macherey-Nagel) or the QIAmp DNA Mini Kit (QIAGEN) and TRIzol reagent (Invitrogen), respectively. To minimize gDNA contamination, RNA isolated with the Trizol reagent was digested with recombinant RNase-free DNase I (Roche) and further purified with the RNeasy kit (QIAGEN). The RNA isolation procedure with NucleoSpin Triprep Kit already includes DNase I treatment.

### Expression analysis by reverse transcription-real time PCR (RT-qPCR)

cDNA synthesis and qPCR were performed using either TaqMan Gene expression assays (Applied Biosystems) or SYBR Green detection as described previously [61], [73]. Primer sequences and TaqMan Gene Expression Assay ID numbers are listed in Tables S1 and S2.

### DNA methylation analysis

Bisulfite conversion of gDNA (0.5–1.5 µg) and PCR reactions were performed as described previously [73]. All PCR amplifications involved semi-nested reactions. Primers (Tables S3 and S4) were designed using MethPrimer [74]. CpG islands were identified with the CpG Island Searcher using default settings [75]. Pyrosequencing reactions were performed by Varionostic GmbH (Ulm, Germany). For bisulfite sequencing, PCR products were subcloned using the StrataClone™ PCR Cloning Kit and StrataCone™ SoloPack® Competent Cells (Agilent Technologies) according to the manufacturer's instructions. Sequences were analyzed with BISMA software using default settings [76].

### Fluorescence activated cell sorting (FACS) analysis

EBs were washed twice in PBS and incubated in Cell Dissociation Buffer (Invitrogen) for 45 min at 37°C. An equal volume of EB medium was added and EBs were dissociated to single cells by vortexing. To distinguish dead from living cells, 7-amino-actinomycin D (Invitrogen) was added at a concentration of 5 µg/ml for 20 min at 4°C. After multiple washing steps in PBS, cells were fixed in Cytofix (BD biosciences) for 20 min at 4°C and washed twice in Perm/Wash solution (BD bioscience). Antibody staining was performed in Perm/Wash buffer for 30 min at 4°C using Alexa Flour® 647 Mouse anti-Oct3/4 and Alexa Flour® 647 Mouse IgG1 K isotype control (BD Biosciences). After washing twice with Perm/Wash buffer, cells were resuspended in Annexin buffer (10 mM HEPES, 140 mM NaCl, 2.5 mM CaCl_2_, pH 7.4) and analyzed with a FACS Aria II instrument (Becton Dickinson). Data analysis was performed using FlowJo version 7.2.5.

### Microarray and statistical analysis

Sample preparation for microarray analysis was performed using the WT Expression Kit (Ambion) and WT Terminal Labeling and Controls Kit (Affymetrix) with 300 ng input RNA. Samples were hybridized to GeneChip Mouse Gene 1.0 ST microarrays (Affymetrix) according to the manufacturer's instructions. Quality control, normalization and further statistical analyses were carried out using the R/Bioconductor programming environment. Linear Models for Microarray Data (limma) was used to compute fold changes and p-values [77]. Genes with a fold change of 2 and a false discovery rate below 0.05 were considered differentially expressed. Genes identified as differentially expressed (Fig. 3B–D) and their expression fold change are listed in Table S5 (d0), S6 (d0–4) and S7 (d4–16). Gene ontology (GO), tissue expression and chromosomal location analysis was performed using DAVID [78], [79]. For GO enrichment analysis the lowest level of GO categories for biological process (GO_BP_ALL) were used. A one-sided Kolmogorov-Smirnov test was used to compare gene expression changes in wt and knockout EBs globally as well as for bivalent and non-bivalent genes between day 0 and 4 of differentiation (Fig. 4 and Fig. S8).

### Accession codes

Microarray data have been deposited into the Gene Expression Omnibus (GEO) database under the accession number GSE36679.

## Supporting Information

Figure S1 **Stable complementation of** ***Dnmt1^−/−^*** **ESCs with GFP-Dnmt1^wt^, but not the catalytically inactive mutant GFP-Dnmt1^C1229W^ rescues silencing and promoter methylation of** ***Oct4*** **and** ***Nanog*** **upon differentiation as EBs.**
(TIF)

Figure S2 **Analysis of genes differentially expressed in TKO ESCs compared to wt ESCs (related to** **Fig. 3B** **).**
(TIF)

Figure S3 **Gene ontology enrichment and cell type specific expression of concordantly regulated genes in wt,** ***Dnmt1^−/−^*** **and TKO EBs after 4 days of differentiation (related to** **Fig. 3C** **).**
(TIF)

Figure S4 **Gene ontology enrichment and cell type specific expression of concomitantly upregulated genes in wt,** ***Dnmt1^−/−^*** **and TKO EBs during day 4–16 of differentiation (related to** **Fig. 3D** **).**
(TIF)

Figure S5 **Enriched GO categories of commonly upregulated genes in wt and** ***Dnmt1^−/−^*** **EBs after 4 days of differentiation (related to** **Fig. 3C** **).**
(TIF)

Figure S6 **Gene ontology enrichment and tissue specific expression of concordantly regulated genes in wt and** ***Dnmt1^−/−^*** **EBs during day 4–16 of differentiation (related to** **Fig. 3D** **).**
(TIF)

Figure S7 **Dnmt3a and 3b transcript leves during EB differentiation of wt and** ***Dnmt1^−/−^*** **ESCs.**
(TIF)

Figure S8 **Expression changes of non-bivalent and bivalent genes in TKO and** ***Dnmt1^−/−^*** **EBs relative to wt EBs between day 0 and 4 of differentiation (related to** **Fig. 4** **).**
(TIF)

Figure S9 **GO and tissue expression enrichment for genes commonly upregulated in** ***Dnmt1^−/−^*** **and TKO EBs as well as exclusively upregulated in** ***Dnmt1^−/−^*** **or TKO EBs after 4 days of differentiation (related to** **Fig. 3C** **).**
(TIF)

Figure S10 **Transcript levels and CpG island methylation of bivalent genes** ***Brachury*** **and** ***Fgf5*** **(related to** **Fig. 5** **).**
(TIF)

Figure S11 **Stable complementation with GFP-Dnmt1^wt^, but not the catalytically inactive mutant GFP-Dnmt1^C1229W^, abolishes reversion of cells from** ***Dnmt1^−/−^*** **EBs to the ESC state upon dissociation and LIF stimulation (related to** **Fig. 6** **).**
(TIF)

Table S1 **TaqMan Assay ID numbers for qPCR.**
(PDF)

Table S2 **Primer Sequences for qPCR.**
(PDF)

Table S3 **Primer Sequences for bisulfite Sequencing.**
(PDF)

Table S4 **Primer Sequences for Pyrosequencing.**
(PDF)

Table S5 **Differentially expressed genes in** ***Dnmt1^−/−^*** **and TKO ESCs (related to** **Fig. 3B** **).**
(XLSX)

Table S6 **Differentially expressed genes between d0–4 of differentiation (related to** **Fig. 3C** **).**
(XLSX)

Table S7 **Differentially expressed genes between d4–16 of differentiation (related to** **Fig. 3D** **).**
(XLSX)
