# Supplementary figures and images for: Global DNA Hypomethylation Prevents Consolidation of Differentiation Programs and Allows Reversion to the Embryonic Stem Cell State
Source: PLoS One. 2012 Dec 27;7(12):e52629. doi: 10.1371/journal.pone.0052629 (PMC3531338; doi:10.1371/journal.pone.0052629)

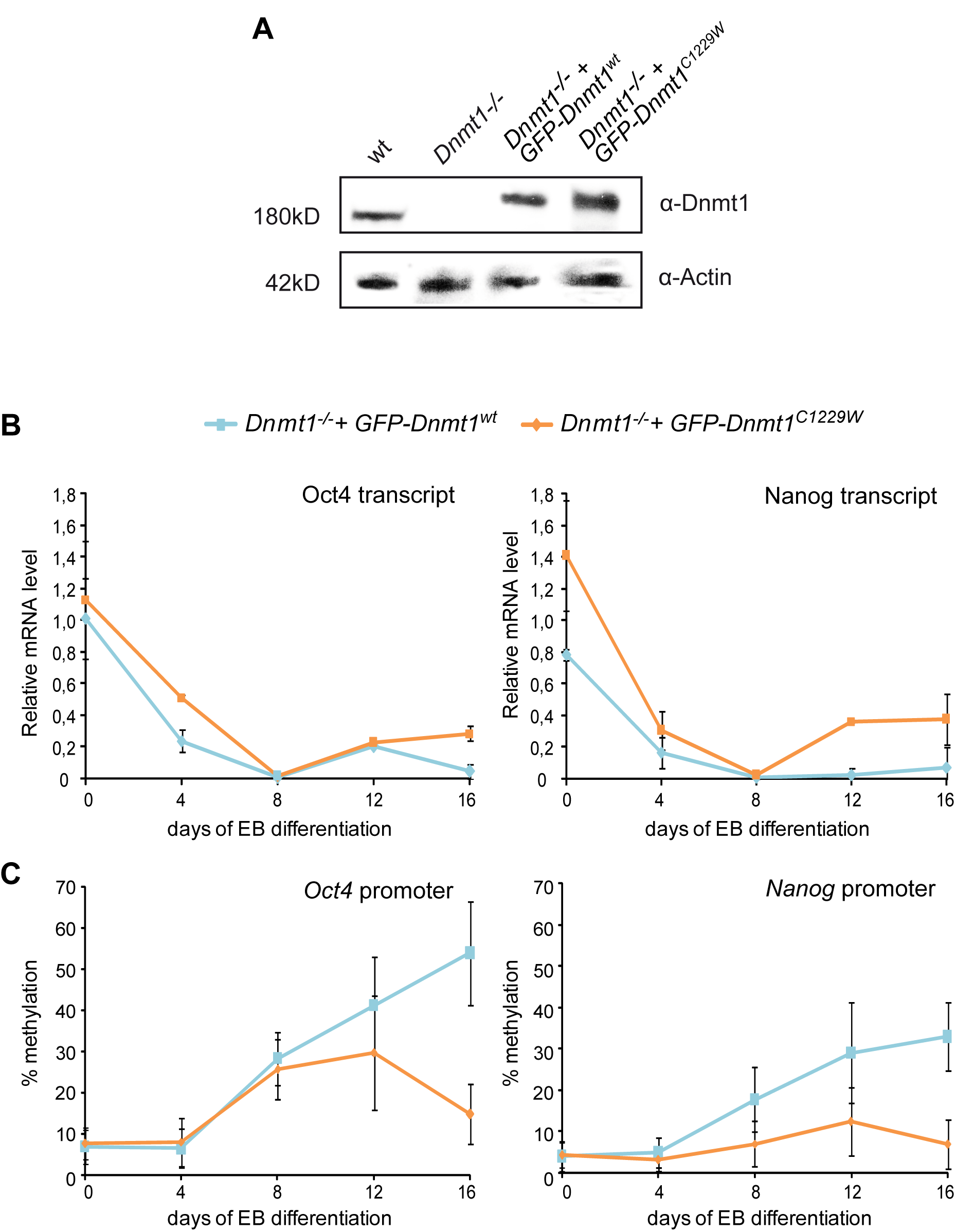

Supplement: Figure S1 — Stable complementation of Dnmt1−/− ESCs with GFP-Dnmt1wt, but not the catalytically inactive mutant GFP-Dnmt1C1229W rescues silencing and promoter methylation of Oct4 and Nanog upon differentiation as EBs. (TIF) [file pone.0052629.s001.tif]

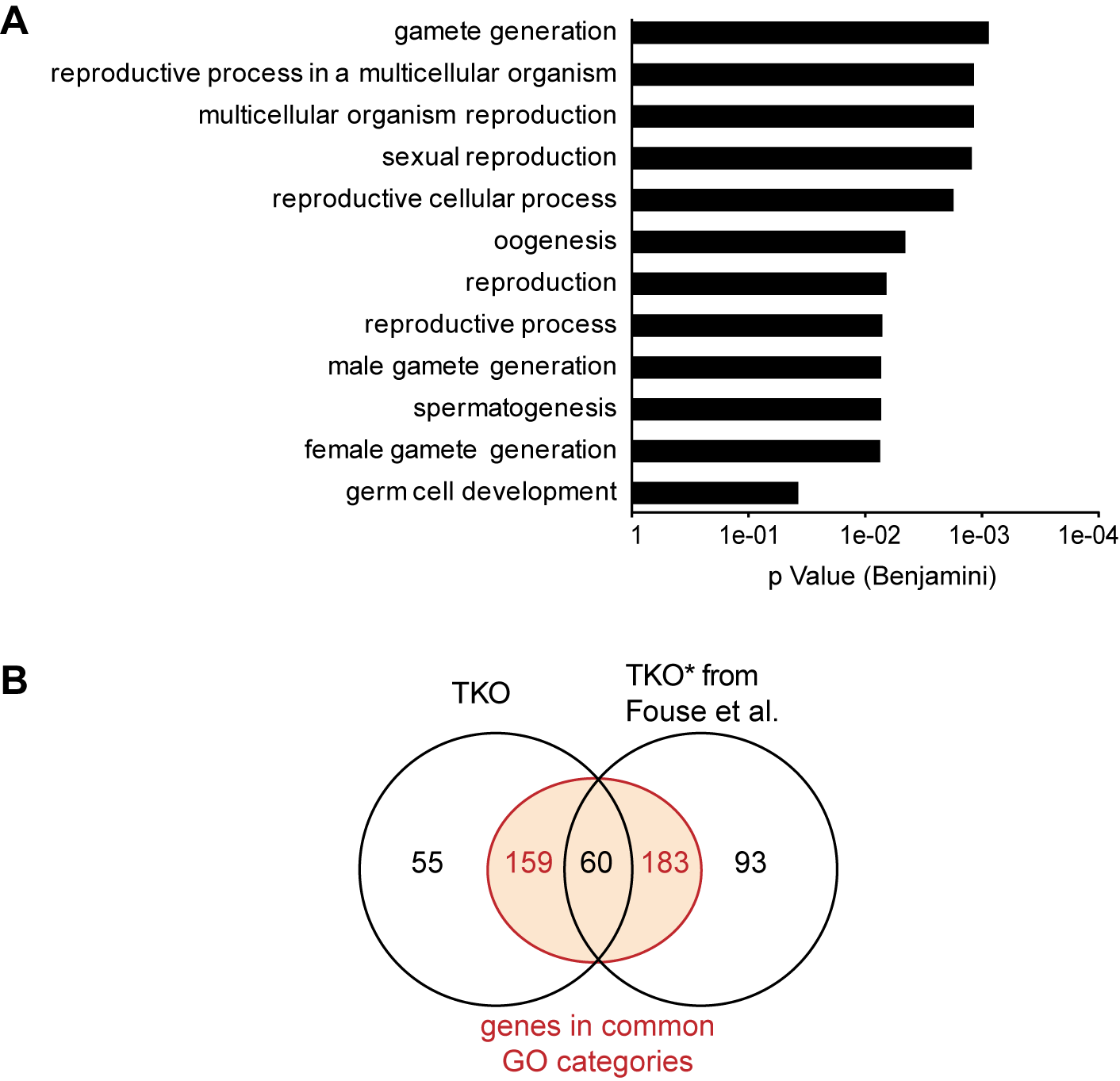

Supplement: Figure S2 — Analysis of genes differentially expressed in TKO ESCs compared to wt ESCs (related to Fig. 3B ). (TIF) [file pone.0052629.s002.tif]

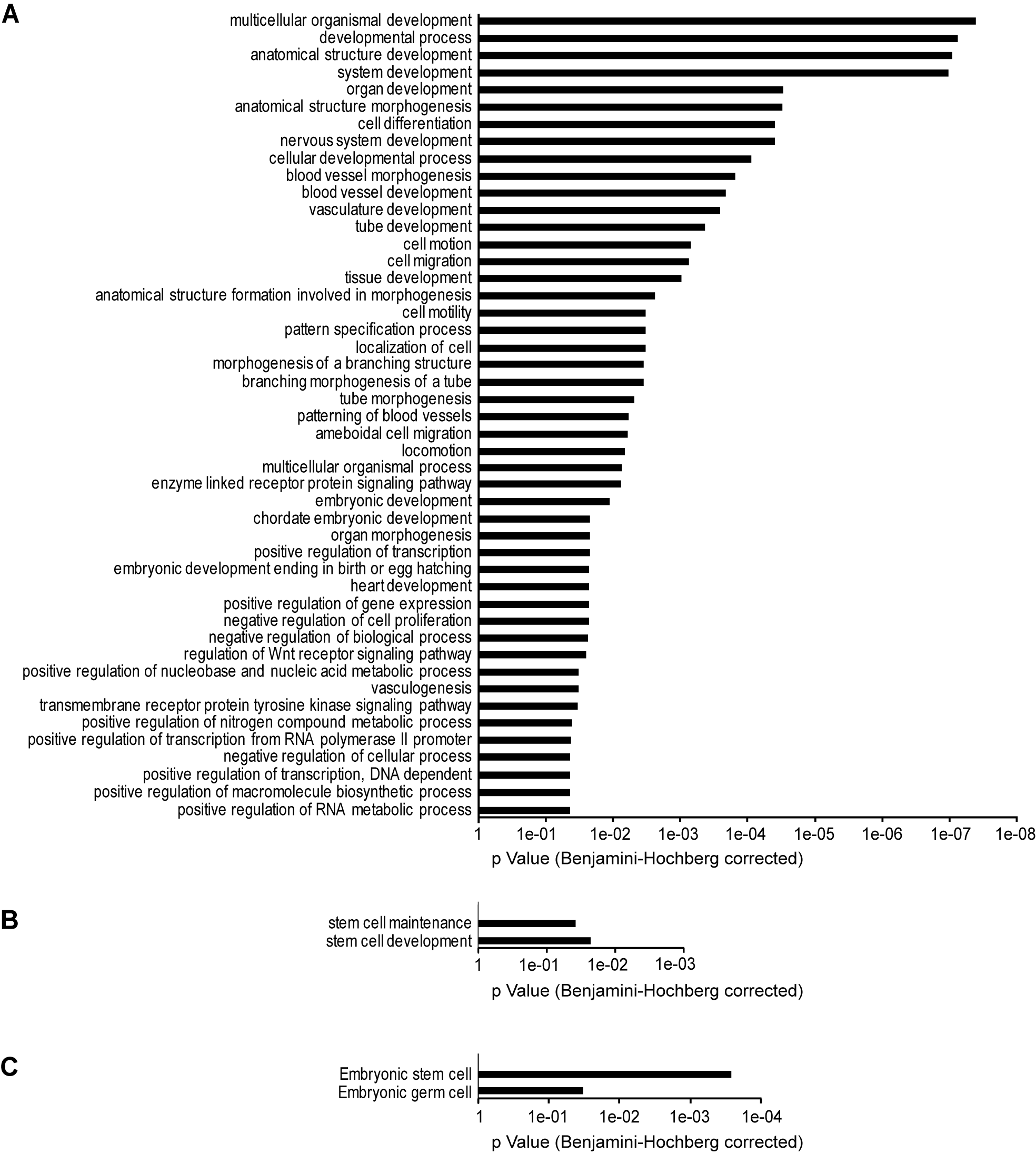

Supplement: Figure S3 — Gene ontology enrichment and cell type specific expression of concordantly regulated genes in wt, Dnmt1−/− and TKO EBs after 4 days of differentiation (related to Fig. 3C ). (TIF) [file pone.0052629.s003.tif]

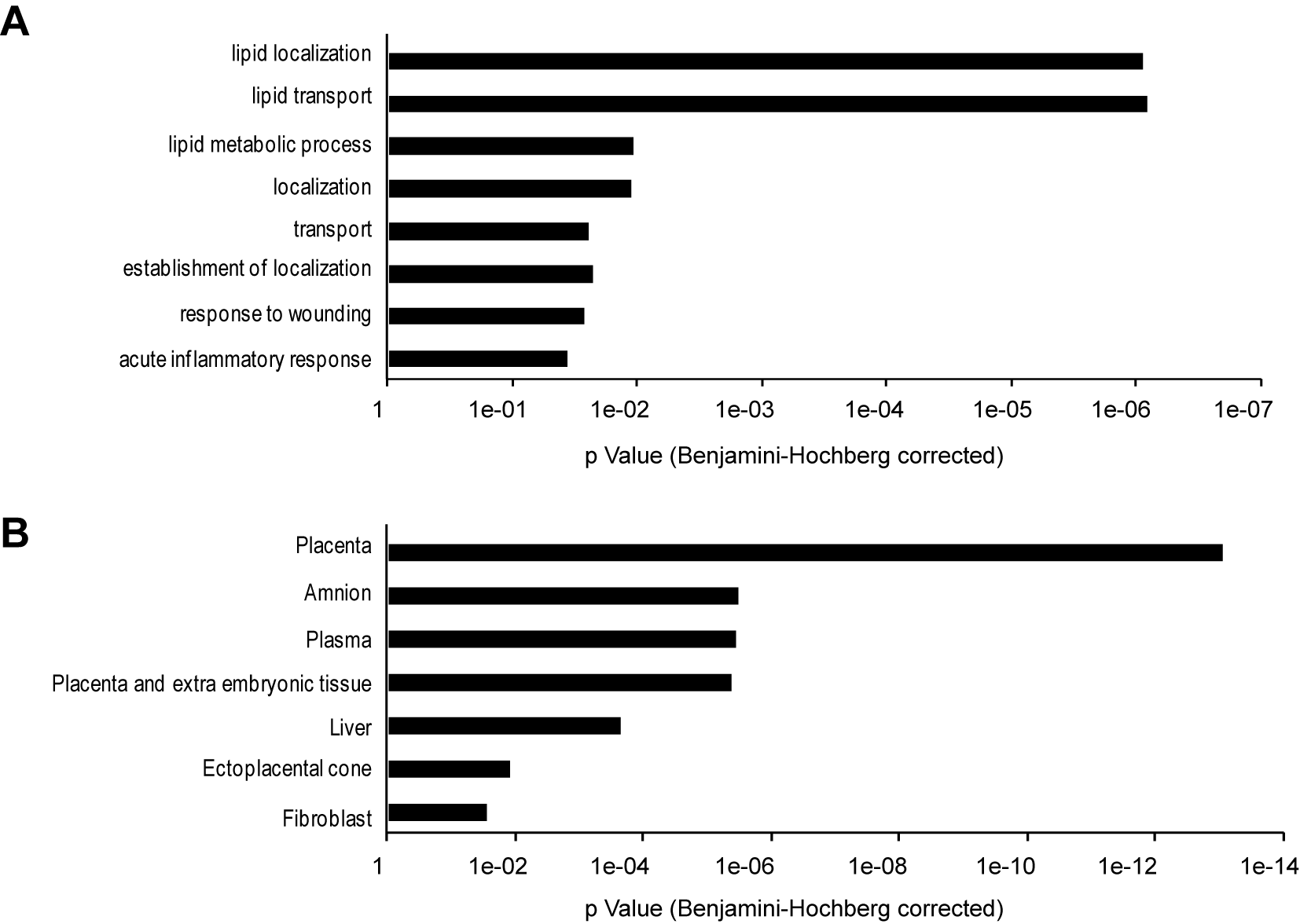

Supplement: Figure S4 — Gene ontology enrichment and cell type specific expression of concomitantly upregulated genes in wt, Dnmt1−/− and TKO EBs during day 4–16 of differentiation (related to Fig. 3D ). (TIF) [file pone.0052629.s004.tif]

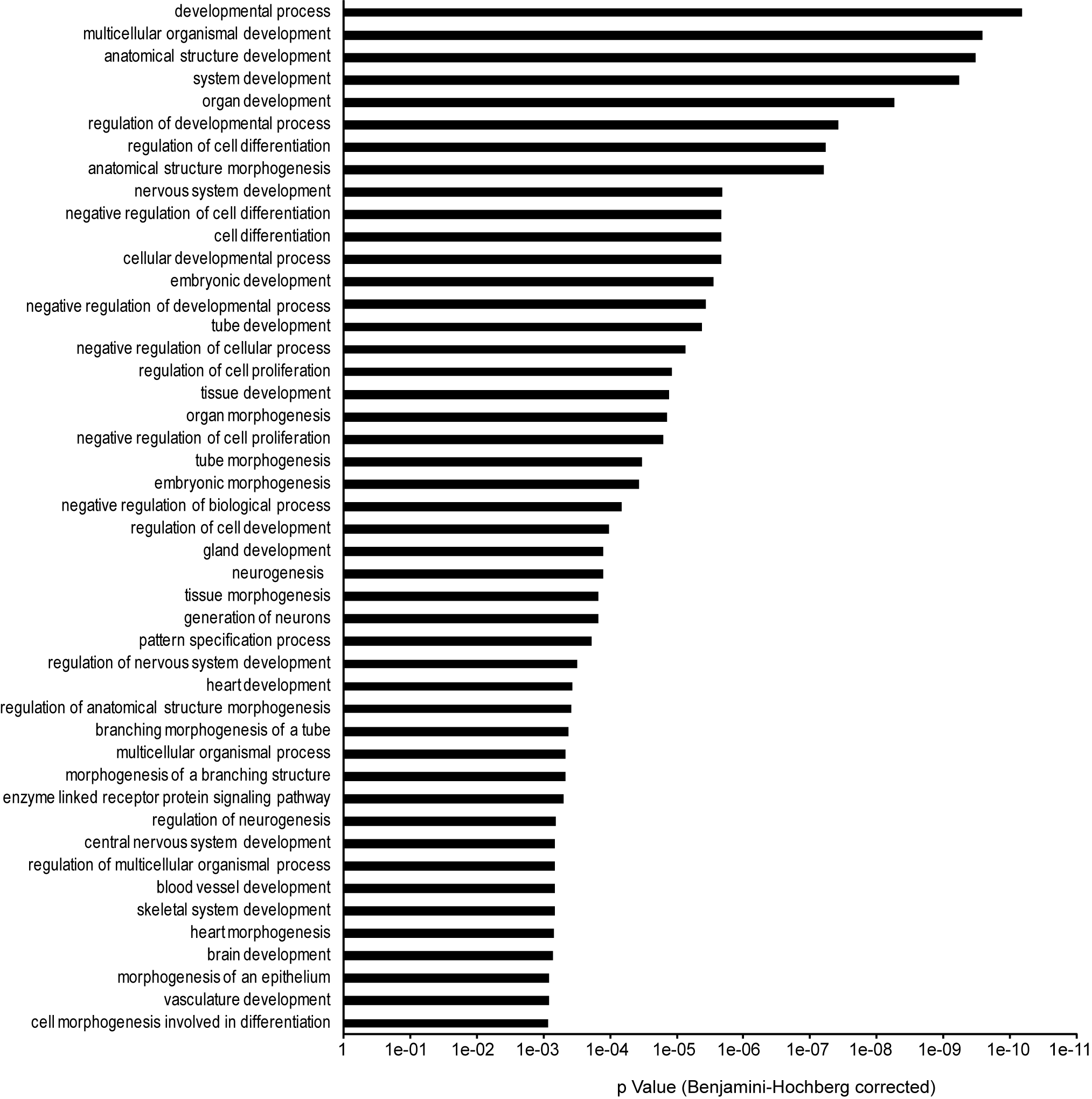

Supplement: Figure S5 — Enriched GO categories of commonly upregulated genes in wt and Dnmt1−/− EBs after 4 days of differentiation (related to Fig. 3C ). (TIF) [file pone.0052629.s005.tif]

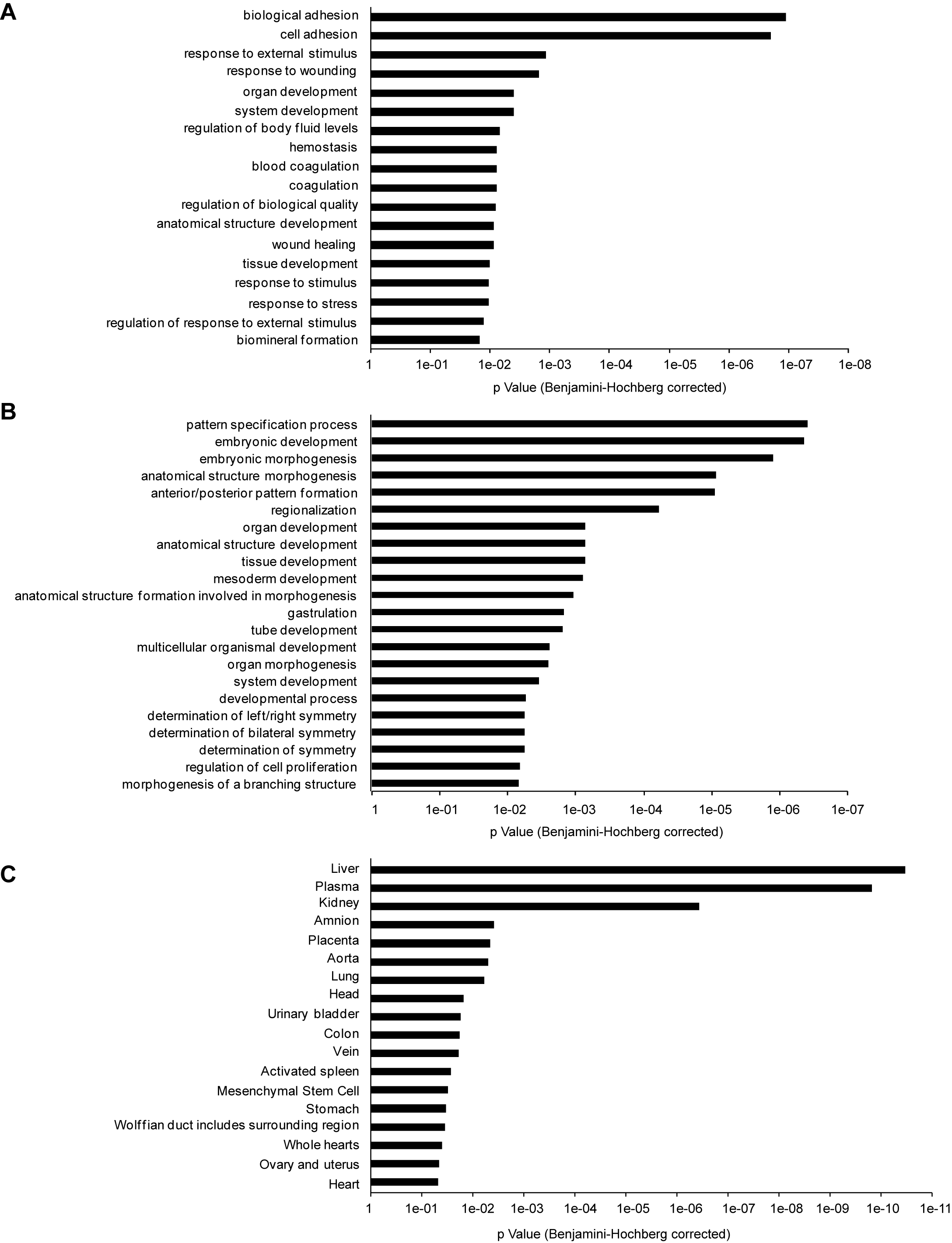

Supplement: Figure S6 — Gene ontology enrichment and tissue specific expression of concordantly regulated genes in wt and Dnmt1−/− EBs during day 4–16 of differentiation (related to Fig. 3D ). (TIF) [file pone.0052629.s006.tif]

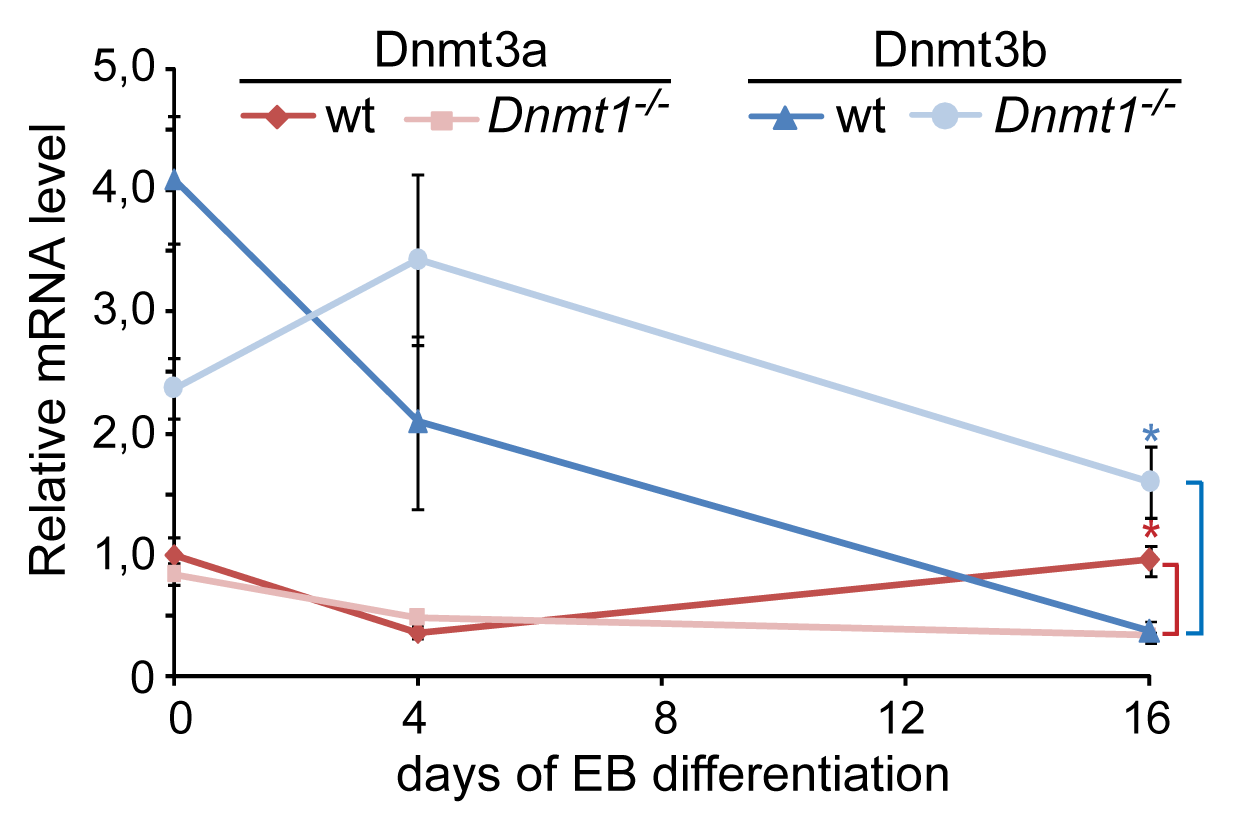

Supplement: Figure S7 — Dnmt3a and 3b transcript leves during EB differentiation of wt and Dnmt1−/− ESCs. (TIF) [file pone.0052629.s007.tif]

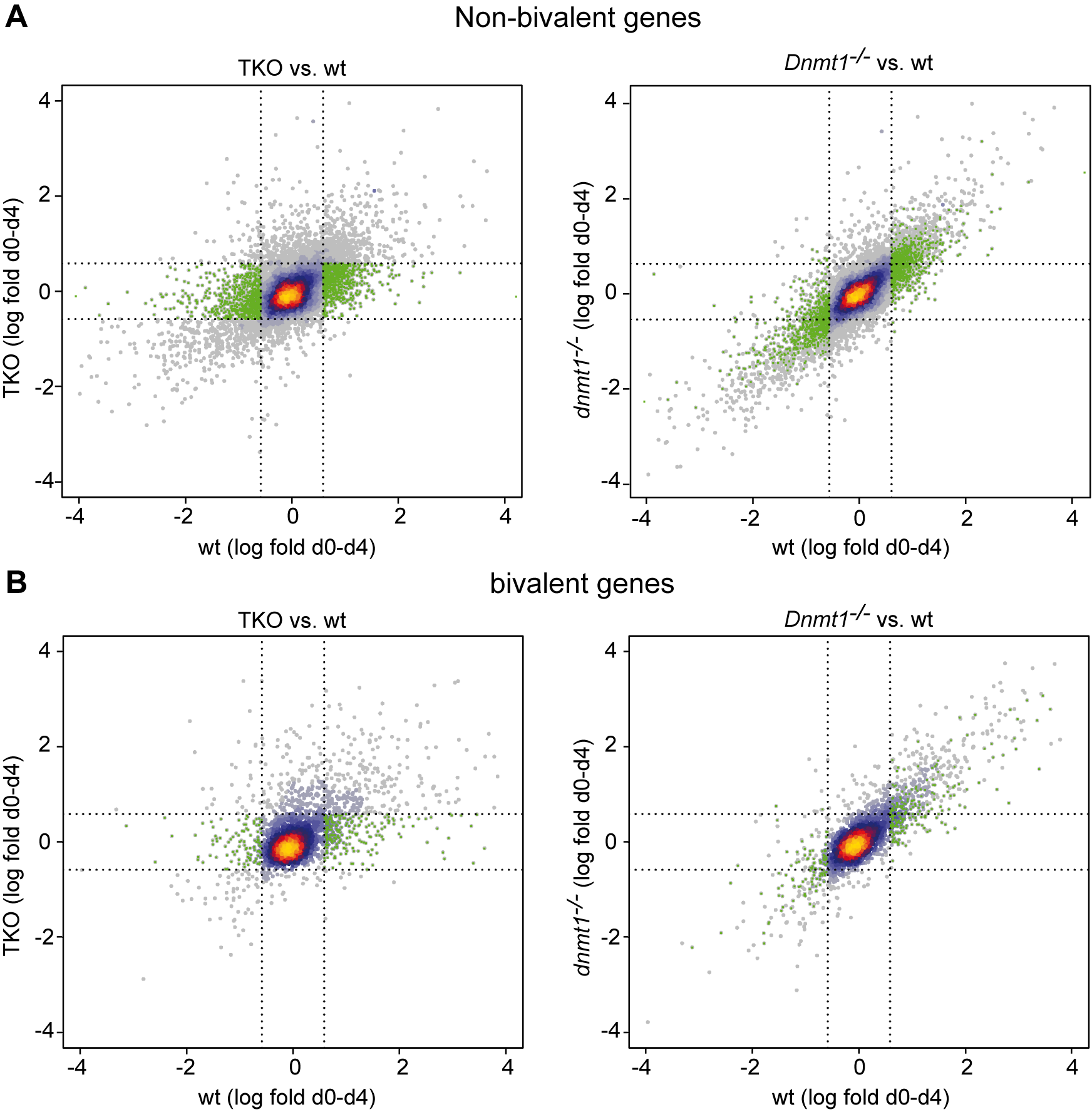

Supplement: Figure S8 — Expression changes of non-bivalent and bivalent genes in TKO and Dnmt1−/− EBs relative to wt EBs between day 0 and 4 of differentiation (related to Fig. 4 ). (TIF) [file pone.0052629.s008.tif]

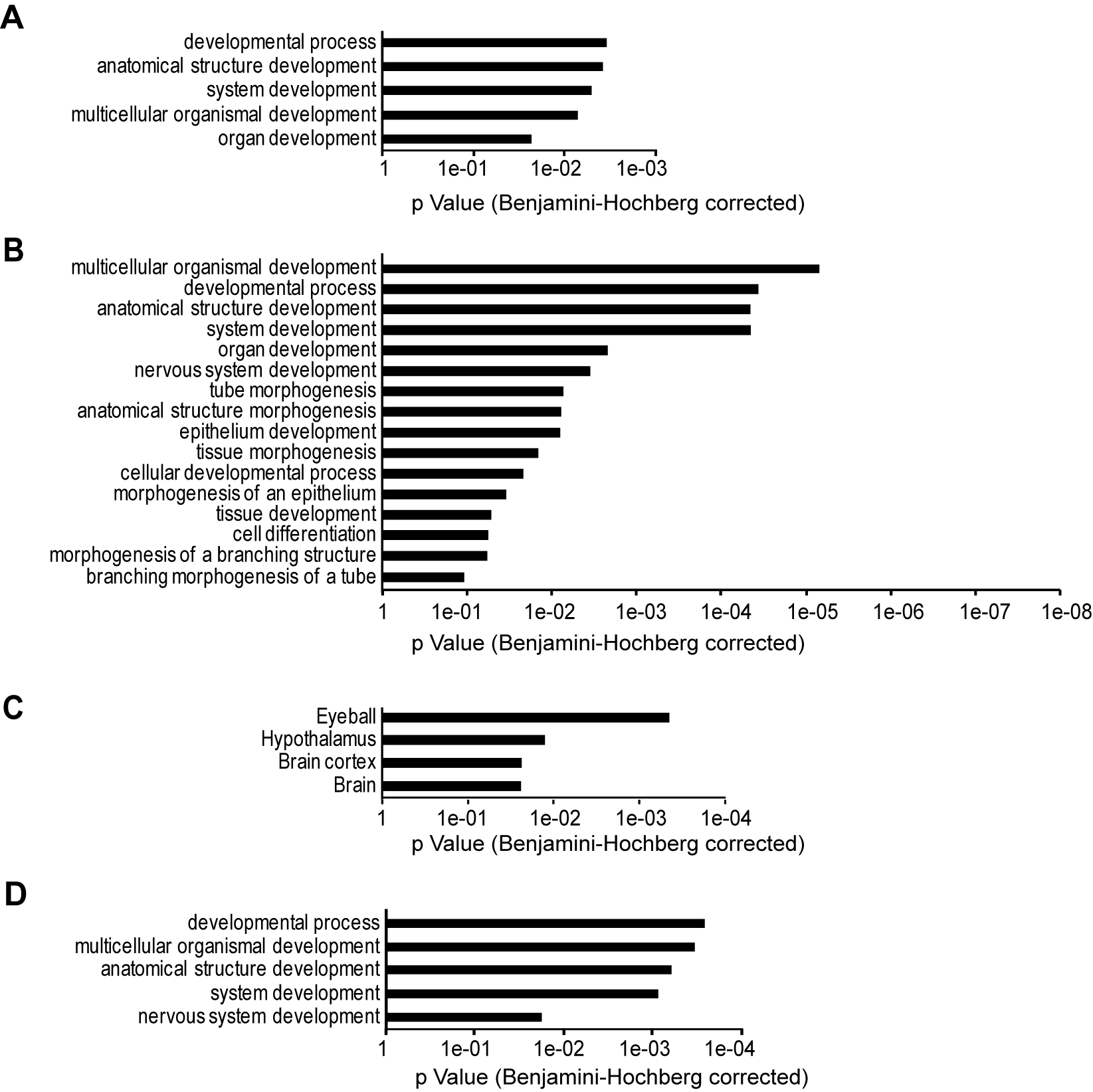

Supplement: Figure S9 — GO and tissue expression enrichment for genes commonly upregulated in Dnmt1−/− and TKO EBs as well as exclusively upregulated in Dnmt1−/− or TKO EBs after 4 days of differentiation (related to Fig. 3C ). (TIF) [file pone.0052629.s009.tif]

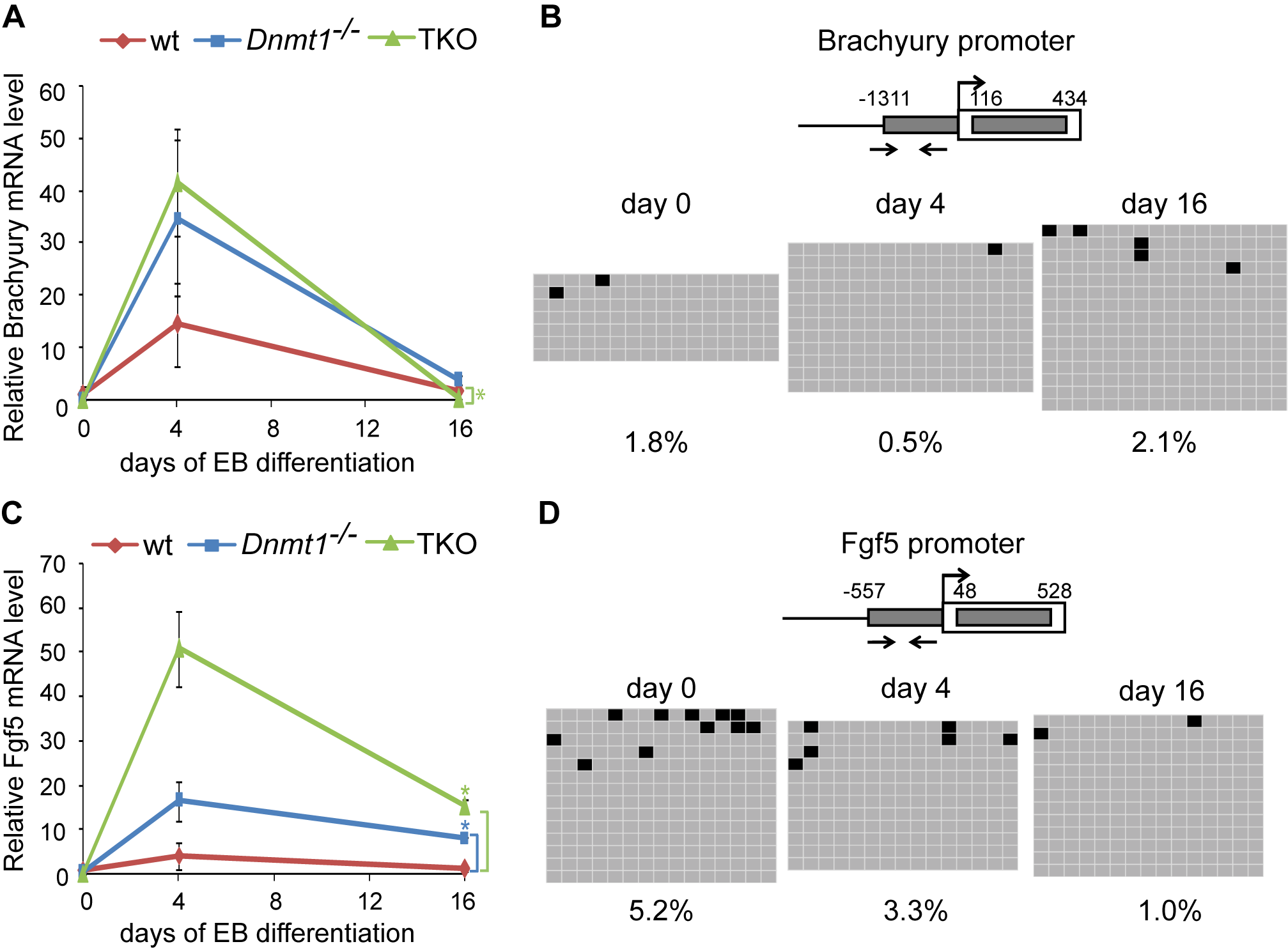

Supplement: Figure S10 — Transcript levels and CpG island methylation of bivalent genes Brachury and Fgf5 (related to Fig. 5 ). (TIF) [file pone.0052629.s010.tif]

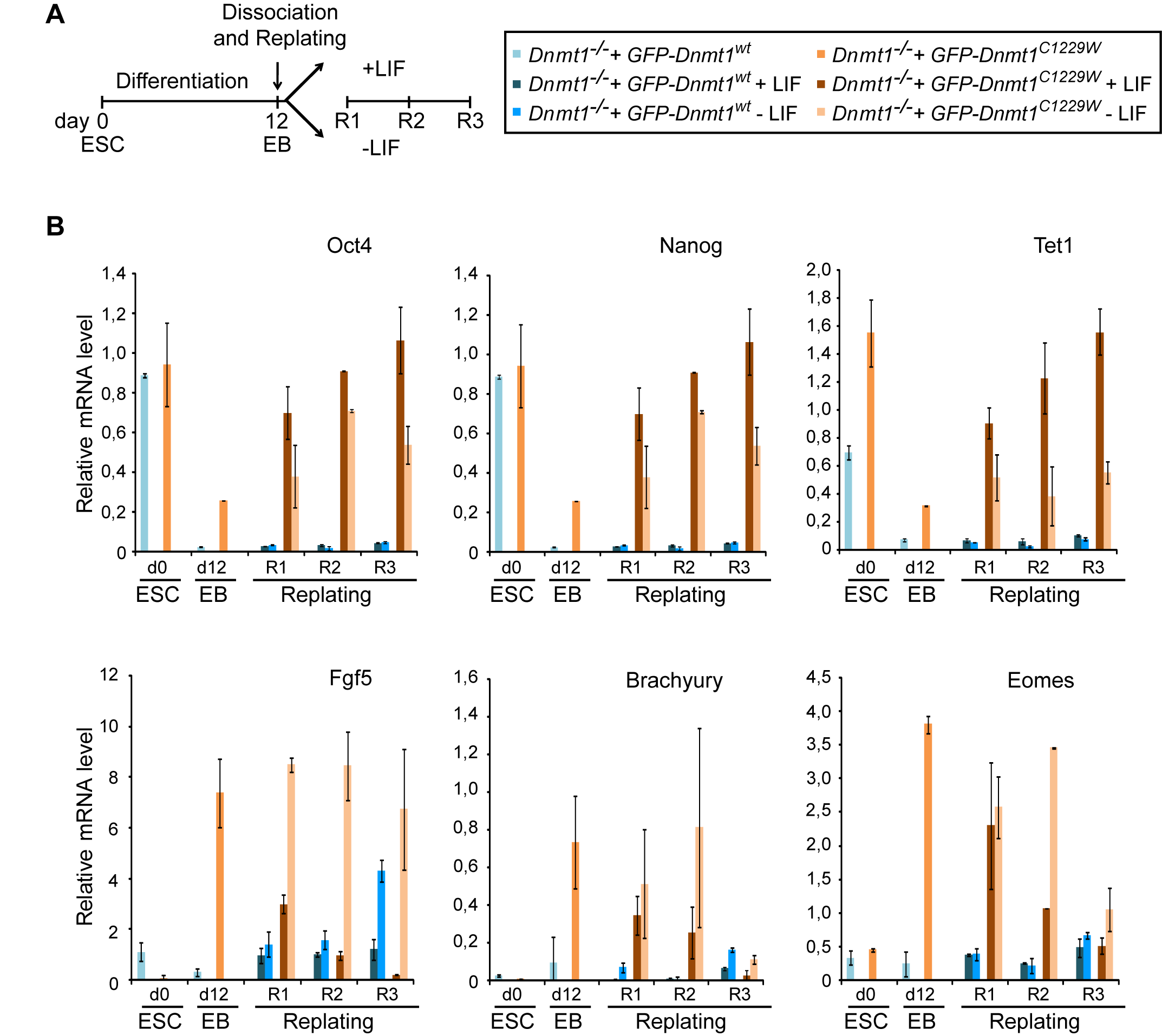

Supplement: Figure S11 — Stable complementation with GFP-Dnmt1wt, but not the catalytically inactive mutant GFP-Dnmt1C1229W, abolishes reversion of cells from Dnmt1−/− EBs to the ESC state upon dissociation and LIF stimulation (related to Fig. 6 ). (TIF) [file pone.0052629.s011.tif]
