# Supplementary material for: Global DNA Hypomethylation Prevents Consolidation of Differentiation Programs and Allows Reversion to the Embryonic Stem Cell State
Source: PLoS One. 2012 Dec 27;7(12):e52629. doi: 10.1371/journal.pone.0052629 (PMC3531338; doi:10.1371/journal.pone.0052629)
Supplement: Table S1 — TaqMan Assay ID numbers for qPCR. (PDF) [file pone.0052629.s012.pdf]

Table S1. TaqMan Assay ID numbers for qPCR

Gene Name	Assay ID
Dnmt3a	Mm00432884_m1
Dnmt3b	Mm01240113_m1
Gapdh	Mm99999915_g1
Oct4	Mm00658129_gH
Nanog	Mm01617761_g1
