# Supplementary material for: Global DNA Hypomethylation Prevents Consolidation of Differentiation Programs and Allows Reversion to the Embryonic Stem Cell State
Source: PLoS One. 2012 Dec 27;7(12):e52629. doi: 10.1371/journal.pone.0052629 (PMC3531338; doi:10.1371/journal.pone.0052629)
Supplement: Table S2 — Primer Sequences for qPCR. (PDF) [file pone.0052629.s013.pdf]

Table S2. Primer Sequences for qPCR

**Gene**	**Forward Primer (5´-3´)**	**Reverse Primer (5´-3´)**
Brachyury	CTC CAA CCT ATG CGG ACA ATT C	ATG ACT CAC AGG CAG CAT GCT
Eomes	ACC GGC ACC AAA CTG AGA TGA	GGG GTT GAG TCC GTT TAT GTT GAA
fgf5	GAT CTA CCC GGA TGG CAA AG	TGC TGA AAA CTC CTC GTA TTC CT
Gapdh	CAT GGC CTT CCG TGT TCC TA	CTT CAC CAC CTT CTT GAT GTC ATC
Gata6	CAA AAG CTT GCT CCG GTA ACA	GGT CGC TTG TGT AGA AGG AGA AG
Nestin	ACT CTG CTG GAG GCT GAG AAC T	CAA GGA AAT GCA GCT TCA GCT T
Sox1	GCT TCG GAG GAC AAA AGA CAA	AAG AGC TGG CGG GAA GTA AAC
Tet1	CCA GGA AGA GGC GAC TAC GTT	TTA GTG TTG TGT GAA CCT GAT TTA TTG T
