# Supplementary material for: Global DNA Hypomethylation Prevents Consolidation of Differentiation Programs and Allows Reversion to the Embryonic Stem Cell State
Source: PLoS One. 2012 Dec 27;7(12):e52629. doi: 10.1371/journal.pone.0052629 (PMC3531338; doi:10.1371/journal.pone.0052629)
Supplement: Table S3 — Primer Sequences for bisulfite Sequencing. (PDF) [file pone.0052629.s014.pdf]

Table S3. Primer Sequences for bisulfite Sequencing

Gene	Forward Primer (5´-3´)	Reverse Primer (5´-3´)
Brachyury (PCR 1)	GGT GGG AGT TAG TGG TAG TTT AT	CAA AAC CCT AAC TCC TAA AAC CA
Brachyury (PCR 2)	TTT AAA GTT GTT ATA TTT GGG GAG GT	= Reverse from PCR1
Fgf5 (PCR1)	TAG GGT GGT TTT TTA GTG GAG AAA T	ATT ATC AAA AAC CAC CCA ATC ACC
Fgf5 (PCR2)	ATG GTA GGG GTT AGT AAT TTG GAA	= Reverse from PCR1
Nestin (PCR1)	GGA AGA ATT TTT TTA GAT GTG GGA G	CAA CCT AAA TAC TCA ACC ACC C
Nestin (PCR2)	GAG GAG TAG AAT TAG TTG TTT AGT	= Reverse from PCR1
Sox1 (PCR1)	GTT AGT TTA GGT TGG GTT TTA TGA AAT	AAT CCC TAT CTC AAA ACC TAC TAC
Sox1 (PCR2)	= Forward from PCR1	CCT AAA CCT ATC AAT ATA AAC CCT AT
Tet1 (PCR1)	TTT TTA GGA TGT TAT TTG GAT GAT T	CAC AAC CTT TAC TAA ACCC TAT ACC
Tet1 (PCR2)	= Forward from PCR1	TAT CTC CCC AAT ACA AAC CTC
