# Supplementary material for: Global DNA Hypomethylation Prevents Consolidation of Differentiation Programs and Allows Reversion to the Embryonic Stem Cell State
Source: PLoS One. 2012 Dec 27;7(12):e52629. doi: 10.1371/journal.pone.0052629 (PMC3531338; doi:10.1371/journal.pone.0052629)
Supplement: Table S4 — Primer Sequences for Pyrosequencing. (PDF) [file pone.0052629.s015.pdf]

Table S4. Primer Sequences for Pyrosequencing

Gene	Forward Primer (5´-3´)	Reverse Primer (5´-3´)
Oct4 (PCR 1)	ATG GGT TGA AAT ATT GGG TTT ATT TA	ACC CTC TAA CCT TAA CCT CTA AC
Oct4 (PCR 2)	GTA AGA ATT GAG GAG TGG TTT TAG	= Reverse from PCR1 with 5´biotinylated
Nanog (PCR1)	TAG TTT GGG TTA TTT TAT AGT TTT TTT TG	CCA AAA AAA CCC ACA CTC ATA TC
Nanog (PCR2)	AAT GTT TAT GGT GGA TTT TGT AGG T	= Reverse from PCR1 with 5´biotinylated
